# Orchestrating T and NK cells for tumor immunotherapy via NKG2A-targeted delivery of a de novo designed IL-2Rβγ agonist

**DOI:** 10.1080/10717544.2025.2482195

**Published:** 2025-04-01

**Authors:** Jie Chen, Enhui Ren, Ze Tao, Hongyu Lu, Yunchuan Huang, Jing Li, Yuzhe Chen, Zhuo Chen, Tianshan She, Hao Yang, Hong Zhu, Xiaofeng Lu

**Affiliations:** ^a^ Division of Abdominal Tumor Multimodality Treatment, Cancer Center; NHC Key Lab of Transplant Engineering and Immunology, Regenerative Medicine Research Center, West China Hospital, Sichuan University, Chengdu, China; ^b^ Sichuan Provincial Engineering Laboratory of Pathology in Clinical Application, West China Hospital, Sichuan University, Chengdu, China; ^c^ Institutes for Systems Genetics, Frontiers Science Center for Disease-Related Molecular Network, West China Hospital, Sichuan University, Chengdu, China; ^d^ Division of Abdominal Tumor Multimodality Treatment, Cancer Center, West China Hospital, Sichuan University, Chengdu, China

**Keywords:** Immunotherapy, immune checkpoint inhibitor, IL-2R agonist, T and NK cell exhaustion, IgG-binding domain

## Abstract

As T and NK cell exhaustion is attributed to increased expression of immune checkpoints and decreased production of proliferative cytokines by these cells, immune checkpoint-targeted delivery of proliferative cytokines might induce robust and sustained antitumor immune responses. Here, the expression profile of NKG2A was first found to be narrower than that of PD-1 in tumor-infiltrated immune cells. Moreover, unlike PD-1, NKG2A was predominantly co-expressed with IL-2Rβγ in tumor-infiltrated CD8^+^ T and NK cells, but not in Tregs, suggesting that NKG2A might be an ideal target for delivery of IL-2Rβγ agonists to overcome T and NK exhausting. For NKG2A-targeted delivery of an IL-2Rβγ agonist, a single molecule of de novo designed N215 endowed with Immunoglobin G(IgG)-binding ability was coupled to an antibody against NKG2A (αNKG2A) to produce αNKG2A-N215. NKG2A- and IL-2Rβγ-binding were well preserved in αNKG2A-N215, allowing αNKG2A-N215 to act as both an immune checkpoint inhibitor and a T and NK cell stimulator. Intravenously injected αNKG2A-N215 predominantly induced expansion of tumor-infiltrated CD8^+^ T and NK cells while showing little stimulation of Tregs. Compared with the separate combination using αNKG2A and N215, αNKG2A-N215 exerted a greater antitumor effect in mice bearing MC38 or B16/F1 tumors. 50% of mice bearing MC38 tumors were cured by αNKG2A-N215, and long-term immunological memory against the tumor was induced in these mice. These results indicate that NKG2A is another ideal target for delivery of an IL-2Rβγ agonist, and αNKG2A-N215, with specificities for both NKG2A and IL-2Rβγ, might be developed as a novel agent for immunotherapy.

## Introduction

Cytotoxic T cells and natural killer (NK) cells play complementary and intertwined roles in tumor recognition and killing, which is essential for human antitumor immunity. However, recent studies have demonstrated that continued exposure to tumor antigens and immunosuppressive tumor environments can induce both T and NK cell exhaustion (Roe, 2022, Jia et al., 2023), which further increases the expression of immune checkpoints including programmed death-1(PD-1), cytotoxic T-lymphocyte associated antigen 4 (CTLA-4), and NK cell receptor 2 A (NKG2A) to reinforce exhaustion of cytotoxic T and NK cells (van Montfoort et al., 2018, Ducoin et al., 2022). Antitumor activities of cytotoxic T and NK cells could be partially normalized by administration of immune checkpoint inhibitors (ICIs) such as antibodies against especially PD-1 or CTLA-4, which have produced unprecedent progression-free and even tumor-free survival in many cancer patients. However, objective response rates to present ICIs are less than 30% in many types of cancer patients, suggesting that more efforts are required to improve the antitumor effect of these ICIs-based immunotherapies (Tang et al., 2022). In addition, it has been found that decreased secretion of proliferative cytokines is extensively observed in exhausted T and NK cells, which resulted in a reduction of immune effector cells in tumors (Propper and Balkwill, 2022), suggesting that combination therapy using ICIs and proliferative cytokines might be more efficient at inducing antitumor immune responses.

Interleukin-2 (IL-2) has been proven as the most robust cytokine that can stimulate proliferation and activation of both cytotoxic T and NK cells overexpressing heterodimeric IL-2 receptor βγ (IL-2Rβγ) (Spolski et al., 2018, Propper and Balkwill, 2022, Boersma et al., 2024), suggesting that IL-2 might be used to improve the antitumor effect of ICIs-based immunotherapies. However, it has been found that the native IL-2 preferentially induces proliferation of regulatory T cells (Tregs) overexpressing heterotrimeric IL-2 receptor αβγ (IL-2Rαβγ) at a low concentration, resulting in suppression on both cytotoxic T and NK cells (Spolski et al., 2018). As preferred Tregs stimulation by native IL-2 is predominantly attributed to its affinity for IL-2 receptor α (IL-2Rα), IL-2 variants with compromised IL-2Rα-binding ability have emerged as novel agents for tumor immunotherapy (Jin et al., 2022, Rybchenko et al., 2023). Usually, these IL-2 variants are produced by either site-directed mutation or IL-2Rα-binding masking (Klein et al., 2017, Ptacin et al., 2021), which fail to completely abrogate IL-2Rα-binding while preserve IL-2Rβγ-binding ability of IL-2. Recently, a novel IL-2 mimic, i.e. Neo 2/15 (designated as N215 in this paper) was produced by de novo synthesis (Silva et al., 2019). Unlike all conventional IL-2 variants, N215 shows no IL-2Rα-binding while preserving IL-2Rβγ-binding and agnostic activity, suggesting that N215 carries lower risk of Tregs stimulation, which greatly triggered our interest in developing N215 as novel IL-2Rβγ agonist to improve the antitumor effect of ICIs-based immunotherapy.

Previous studies have demonstrated that the expression level of PD-1 on cytotoxic T cells in tumor tissues is greater than that of PD-1 on cytotoxic T cells in peripheral blood (Xu et al., 2021, Prodi et al., 2024), suggesting that the payload cytokine would preferentially be delivered to tumor-infiltrated cytotoxic T cells by using an antibody against PD-1 as a directional molecule, which would decrease the risk of inducing systemic immune activation. In fact, IL-2 variants fused to antibodies against PD-1 have induced greater antitumor immune responses without inducing systemic immune activation (Codarri Deak et al., 2022, Piper et al., 2023, Tichet et al., 2023). Interestingly, many studies have demonstrated that the level of NKG2A expression in intratumoral cytotoxic T and NK cells is also higher than that in peripheral blood of patients with liver cancer, head and neck squamous cell carcinoma, esophageal cancer, gastric cancer, ovarian cancer, cervical cancer, or breast cancer (André et al., 2018, van Montfoort et al., 2018, Abd Hamid et al., 2019, Sun and Sun, 2019, Rethacker et al., 2022). In addition, according to the single-cell sequencing data from colon cancer (Lee et al., 2020), breast cancer (Kim et al., 2022), and stomach cancer (Kang et al., 2022), we found that the level of NKG2A expression in intratumoral cytotoxic T and NK cells was comparable to that of PD-1. However, unlike PD-1 that was also expressed in other immune cells including Tregs, expression of NKG2A was tightly restricted to cytotoxic T and NK cells, suggesting that NKG2A might be a novel target for the delivery of an IL-2βγ agonist to orchestrate both cytotoxic T and NK cells for tumor immunotherapy.

In this paper, the potential of NKG2A as a novel target for delivery of an IL-2βγ agonist was firstly evaluated by analyzing the expression profile of NKG2A and IL-2Rβγ in human and mouse tumor tissues. Subsequently, a bifunctional protein αNKG2A-N215 was produced by coupling N215 to an antibody against NKG2A (αNKG2A). Finally, the antitumor effect and impact on T and NK cell exhaustion of αNKG2A-N215 were determined and compared to those of αNKG2A and N215 combination therapy. Our results demonstrated that monotherapy with αNKG2A-N215 effectively orchestrated cytotoxic T and NK cells and thus exerted a greater antitumor effect than that achieved by the combination therapy of αNKG2A and N215.

## Materials and methods

### Cell culture and cytotoxicity assays

The murine colon adenocarcinoma cells (MC38), melanoma cells (B16/F1), T cells (CTLL-2) and human natural killer cells (NK92) were obtained from American Type Culture Collection (ATCC, VA). These cells were cultured in RPMI 1640 or DMEM supplemented with 10% fetal bovine serum (FBS) and 1% penicillin-streptomycin (Gibco, NY). For IL-2-dependent CTLL-2 and NK92 cells, murine IL-2 or human IL-2 (200 U/mL, Sino Biological Inc., Beijing, China) was added into the medium (Martinez et al., 2024).

Cytotoxicity of T cells in MC38 cells was measured by a co-culture system according to the description by Li et al. (2024) and Heemskerk et al. (2021) with some modification. Briefly, T cells (CD45^+^CD3^+^) were isolated from the spleen of mice bearing MC38 after treatment with αNKG2A-N215 by fluorescence-activated cell sorting performed on BD FACA Discover S8 (BD, NJ). MC38 (1 × 10^4^/well) were co-cultured with T cells at a ratio of 1:10 for 24 h in the presence of complex of N215-C_Fab_3 (1.3 nM) with αNKG2A or nonspecific IgG (nsIgG). Apoptosis was measured by using Cellevent^TM^ Caspase3/7 (Thermo, CA) according to the descriptions by Tanpure et al. (2017).

### Protein expression and purification of N215-C_Fab_3

To couple the IL-2Rβγ agonist N215 (Silva et al., 2019) to an Immunoglobin G (IgG) against NKG2A, C_Fab_3 containing three tandemly repeated IgG-binding domain (IgBD) (Unverdorben et al., 2015) was genetically fused at the C-terminus of N215 to produce N215-C_Fab_3. The gene encoding N215-C_Fab_3 was subcloned into the pQE30 plasmid at *Bam*HI and *Sal*I followed by transformation into *E. coli* M15 cells. To induce the expression of protein, isopropyl β-D-thiogalactoside (IPTG, 0.1 mM) was added into the culture medium followed by incubation at 25 °C overnight. Cell pellets were resuspended in lysis buffer (50 mM phosphate, pH 8.0, 300 mM NaCl, 20 mM imidazole, and 10 mM β-mercaptoethanol) and lysed using a high-pressure homogenizer (60–70 MPa). Recombinant proteins with an N-terminal 6His-tag were recovered from the bacterial supernatant by using Ni-NTA affinity chromatography (Qiagen, CA). The purity of the proteins was assessed by sodium dodecyl sulfate-polyacrylamide gel electrophoresis (SDS-PAGE) and size exclusion chromatography (SEC) according to our previous descriptions (Fan et al., 2021). Protein concentration was measured by using a BCA assay kit (Thermo, CA) according to the manual provided by the manufacturer. The purified proteins were dialyzed overnight in phosphate-buffered saline (PBS, 10 mM Na_2_HPO_4_, 137 mM NaCl, 2.68 mM KCl, and 2 mM KH_2_PO_4_, pH 7.4) for further use. N215 was prepared in the same way.

### IgG-binding assays

To determine whether the fused Fab-binding domain endowed N215 with IgG-binding ability, N215-C_Fab_3 was mixed with IgG antibody including the rat IgG against murine NKG2A (αNKG2A, BioXcell, NH) and the human IgG against human NKG2A (Monalizumab, Selleck, TX) at different molar ratios and incubated at room temperature for 30 min, followed by separation on size exclusion chromatography (SEC) column (Superdex increase 200 10/300 GL, GE Healthcare, Uppsala, Sweden) with PBS as eluent at a constant flow rate of 0.5 mL/min. N215 was used as a control. To obtain the complex of N215-C_Fab_3 and IgG, proteins showing retention volume that was smaller than that of IgG on the same SEC column were collected.

To measure the affinities of N215-C_Fab_3 for IgG antibodies including αNKG2A and Monalizumab, biolayer interferometry was performed on the Octet RED96E (Pall ForteBio LLC, CA) according to our previous descriptions (Fan et al., 2021). Briefly, IgG antibodies (2 μM, 250 μL) were immobilized onto a protein A-coated sensor (Pall ForteBio LLC, CA) followed by immersion into N215-C_Fab_3 dissolved in PBS supplemented with 0.05% Tween-20 (PBST) at different concentrations (31.5–500 nM) for association and disassociation. The kinetic constants including the association constant (ka), dissociation constant (kd) and affinity constant (KD, KD = kd/ka), were calculated using data analysis software according to a 1:1 binding model.

To investigate whether the bound αNKG2A or Monalizumab would be disassociated from N215-C_Fab_3 by IgGs in serum under *in vitro* conditions, pulldown experiments were performed at first. Antibodies were labeled with biotin according to our previous work (Fan et al., 2021). Subsequently, the complexes of biotin-labeled antibodies and N215-C_Fab_3 were added into mouse serum and incubation at 37 °C. Pulldown was performed using streptavidin-NTA (GE Healthcare, Uppsala, Sweden) after incubation at different time points. N215-C_Fab_3 and antibody in the pulldown products were visualized by western blots with anti-HIS-PE (for N215-C_Fab_3 detection, Bio Legend, CA) and streptavidin-HRP (for antibody detection, Bio Legend, CA), respectively. Streptavidin-NTA-mediated pulldown of N215-C_Fab_3 mixed without biotin-labeled antibody was used as control.

To investigate whether the bound αNKG2A would be disassociated from N215-C_Fab_3 by endogenous IgGs under *in vivo* conditions, time-dependent blood clearances of αNKG2A and N215-C_Fab_3 in their complexes were measured in mice. The complexes of αNKG2A and biotin-labeled N215-C_Fab_3 were intravenously injected into C57/BL6 mice (*N* = 3). Blood samples were collected via the tail vein at different time postinjection. Residual αNKG2A and N215-C_Fab_3 in the blood were measured using enzyme-linked immunosorbent assays (ELISA) with anti-Rat IgG2b (for αNKG2A detection) or Streptavidin-HRP (for N215-C_Fab_3 detection).

### Affinity for NKG2A and IL-2 receptor

Biolayer interferometry performed on the Octet RED96E with corresponding biosensors (Pall ForteBio LLC, CA) was used to measure the affinity of proteins for NKG2A and IL-2R. The murine NKG2A/CD94 with Fc was immobilized onto protein A-coated biosensors for NKG2A-binding assays. Murine IL-2Rβ or human IL-2Rβγ with a 6His-tag (Sino Biological Inc., Beijing, China) were immobilized onto Octet^®^ anti-HIS (HIS2) biosensors for IL-2R-binding assays. The binding of αNKG2A, N215-C_Fab_3, and their complexes to murine NKG2A/CD94 and murine IL-2Rβ was measured by the same methods for IgG-binding. The binding of N215-C_Fab_3 and its complexes with Monalizumab to human IL-2Rβγ was also measured.

### Cell-binding assays

To measure the cell-binding, N215-C_Fab_3 was labeled with 5-carboxynaphthofluorescein (FAM, Sigma, CA) according to our previous works (Fan et al., 2021, Yang et al., 2023). Briefly, the pH of the protein solution was adjusted to pH 8.0 by 10 mM NaHCO_3_ (Sigma, MO). Subsequently, the proteins were incubated with FAM at a molar ratio of 1:10 for 2 h at room temperature in the darkness. The free dyes were removed by using SEC with Sephadex^™^ G-25 (GE Healthcare, Uppsala, Sweden) or dialysis against PBS. Subsequently, the complex of FAM-labeled N215-C_Fab_3 (100 nM) and αNKG2A were incubated with murine peripheral CD8^+^ T cells and NK cells. The bound proteins were measured by flow cytometry. To determine the NKG2A and IL-2βγ-dependent binding of the protein, cells were preincubated with αNKG2A (250 μg) or N215-C_Fab_3 (37.5 μg) for 1.5 h at room temperature prior to adding the complex of αNKG2A and FAM-labeled N215-C_Fab_3. The cells were washed with PBS containing 0.5% FBS and analyzed by flow cytometer BD LSRfortessa (BD, NJ).

### Cell proliferation assays

IL-2-dependent human NK92 and murine CTLL-2 were used to analyze the proliferation ability of proteins according to the descriptions by Cho et al. (2023) and Deuse et al. (2021) with some modifications. Briefly, cells were washed twice with PBS prior to inoculation into 96-well plates (5 × 10^3^ cells/well). Different concentration (0–20 nM) of N215-C_Fab_3 or its complexes with αNKG2A (for CTLL-2) or Monalizumab (for NK92) were added into the cells. After culture at 37 °C for 48 h, the viable cells were measured by using Cell Counting Kit-8 (CCK-8, MCE, NJ) according to the manual provided by the manufacturer. Cell proliferation index was calculated according to the ratio of viability of cells treated with N215-C_Fab_3 to that of cells treated without N215-C_Fab_3.

### Cellular distribution assays

To investigate whether N215-C_Fab_3 and antibody in their complexes would show similar cellular distribution, N215-C_Fab_3 and αNKG2A or Monalizumab were labeled with 5-carboxynaphthofluorescein (FAM, Sigma, CA) or Alexa Fluor 647 (Thermo, CA), respectively. To examine the cellular distribution under *in vitro* conditions, the complexes of N215-C_Fab_3 and Monalizumab were incubated with NK92 cells at room temperature for 30 min. Hochest 33342 (Beyotime, Shanghai, China) was used to visualize nuclei followed by observation under confocal microscopy (Leica, Wetzlar, Germany). For *in vivo* cellular distribution assays, the complexes of N215-C_Fab_3 and αNKG2A were intravenously injected into mice bearing MC38 tumor grafts. About 4 h later, the tumor grafts were collected for frozen section. The cellular distribution of proteins was demonstrated using confocal microscopy after visualizing nuclei with 4′,6-diamidino-2-phenylindole (DAPI, Beyotime, Shanghai, China).

### Animal models and treatments

MC38 cells (1 × 10^6^ cells in 100 μL PBS) or B16/F1 cells (5 × 10^4^ cells in 100 μL PBS) were subcutaneously implanted in C57BL/6 mice (female, 6 weeks old, 16-18 g, *N* = 40 for MC38, *N* = 28 for B16/F1), respectively. Tumor growth was monitored by measuring the length (L) and transverse (W) diameters of tumor grafts, followed by calculating the tumor volume (V) according to the following formula: V = L × W^2^/2. (Overdijk et al., 2020). When the average tumor volume reached 100-150 mm^3^, the mice were randomly divided into four groups (*N* = 10 for MC38, *N* = 7 for B16/F1) for treatment with PBS, the mixture of αNKG2A (129 μg) and 12 μg N215 (αNKG2A + N215 (12 μg)), the complex containing αNKG2A (129 μg) and 12 μg N215 equivalent of N215-C_Fab_3 (αNKG2A-N215 (12 μg)) or the complex containing αNKG2A (43 μg) and 4 μg N215 equivalent of N215-C_Fab_3 (αNKG2A-N215 (4 μg)). After treatment, the tumor growth was monitored every day. Once the tumor volume exceeded 1500 mm^3^, the mice were sacrificed and recorded as dead. Immune cells including CD8^+^ T cells, NK cells and Tregs in peripheral blood as well as in tumor were collected at different times posttreatment for flow cytometry analysis. To determine whether the treatment induced long-term immunological memory, the cured mice were rechallenged by the same types of tumor cells with naïve mice as control. Dynamic change of memory T cells in the blood of rechallenged mice was monitored by flow cytometry analysis.

### Phenotype analysis of peripheral and tumor-infiltrated immune cells of mice

Peripheral blood mononuclear cells (PBMCs) and single-cell suspension of tumor tissues were prepared according to the description by Xu et al. (2021) with some modifications. For PBMCs isolation, the peripheral blood was collected via cardiocentesis or by lateral tail vein after the mice were anesthetized using tribromoethanol. The red blood cells were lysed using red blood cell lysis buffer (YEASEN, Shanghai, China) according to the manual provided by the manufacturer. To prepare tumor single-cell suspension, tumor grafts were collected and weighed at first. Subsequently, tumor tissues were cut into small pieces followed by digestion with collagenase IV (200 U/mL, Gibco, NY) and DNase (10 U/mL, Thermo, CA) dissolved in RPMI 1640 at 37 °C with shaking for 1–1.5 h. After filtration the digested tissues with a 70 μm cell strainer, single-cells were collected for further red blood cell lysis. At last, the remaining single-cells were resuspended in PBS containing 0.5% FBS and stored at 4 °C for flow cytometry analysis.

All antibodies used for flow cytometry were purchased from Bio Legend (CA). Fc receptors (FcRs) ubiquitously expressed on immune cells were blocked with TruStain FcX^™^ PLUS containing anti-mouse CD16/32 to prevent IgG Fc-directed nonspecific binding for all flow cytometry analysis. In some experiments, dead cells were indicated by staining with Live/Dead zombie (Bio Legend, CA) according to the manual provided by the manufacturer. PBS containing 0.5% FBS was used as cell wash buffer. For surface marker staining, cells were incubated with antibodies at room temperature for 30 min in darkness. To examine the intracellular FoxP3, cells were fixed and permeabilized using FoxP3 staining kit (Invitrogen, CA) according to the manufacturer’s instruction followed by staining with antibody against FoxP3 at room temperature for 2 h or at 4 °C overnight. In detail, to determine the expression of NKG2A in CD8^+^ T and NK cells, antibodies including CD45-BV510, CD3-Percp cy5.5, CD8-PE, NK1.1-FITC, and NKG2A-PE cy7 were simultaneously added into cells. To examine the co-expression of IL-2Rβ and NKG2A in CD8^+^ T and NK cells, antibodies including CD45-BV510, IL-2Rβ-Percp cy5.5, CD8-PE, NK1.1-FITC, and NKG2A-PE cy7 were used. To monitor the expression of granzyme B (GzmB) and CD25 in CD8^+^ T, NK cells, and Tregs, antibodies including CD45-BV510, CD3-Percp cy5.5, CD8-PE, NK1.1-FITC, FoxP3-BV421, CD25-BV650, and GzmB-APC were used. CD25^+^Foxp3^+^ T cells were defined as Tregs in these assays (Jeyamogan et al., 2023, Valencia and Lipsky, 2007). Antibodies including CD45-BV510, CD3-Percp cy5.5, CD8-PE, CD4-FITC, CD44-BV421, and CD62L-APC were used for memory T cells analysis. CD44^+^CD62L^+^ T cells and CD44^+^CD62L^-^ T cells were defined as central memory (Tcm) and effector/memory T cells, respectively (Chen et al., 2020). Samples were analyzed on BD LSRfortessa (BD, NJ) or Cytek^®^ Aurora CS (Cytek^®^, CA) or BD FACADiscover S8 (BD, NJ). Data were processed using Flow jo 10.1 software and GraphPad Prism v8 software.

### Expression profiles of NKG2A and PD-1 in tumor-infiltrated immune cells of cancer patients

The single-cell transcriptome datasets of breast cancer (GSE167036) (Liu et al., 2022), colon cancer (GSE132465) (Lee et al., 2020), gastric cancer (GSE206785) (Kang et al., 2022), and melanoma (GSE13982) (Durante et al., 2020) involved in this study were downloaded from the GEO database. Seurat (Version 5.1.0) (Hao et al., 2024) based on R (Version 4.4.1) was applied for subsequent analysis. Cells with ≤ 500 or ≥ 1800 genes/cell were excluded, and ≤ 500 or ≥ 6000 unique molecular identifiers (UMI) per cell were also excluded. After data quality control and standardization, dimensionality reduction, and clustering, cell clusters were identified. Differentially expressed genes of each category were identified by FindMarkers function for Wilcoxon rank sum test, with both min.pct and logfc. thresholds set to 0.25. Due to the focus of this study on the changes of immune cells in the tumor microenvironment, we applied subset function to isolate protein tyrosine phosphatase receptor type C(PTPRC) positive cell populations, and then repeated the above steps to perform detailed clustering of immune cells identified by classical makers (Azizi et al., 2018, Zilionis et al., 2019).

### Statistical analysis

One-way ANOVA was performed using Prism software (GraphPad) for comparison in different groups. The results were expressed as the mean ± standard error of the mean (SEM). The significance was defined as *P* < 0.05 (*), *P* < 0.01 (**), and *P* < 0.001 (***). *P* < 0.0001 (****).

## Results

### NKG2A exhibits a narrower expression profile in tumor-infiltrated immune cells of cancer patients

**Figure 1.**
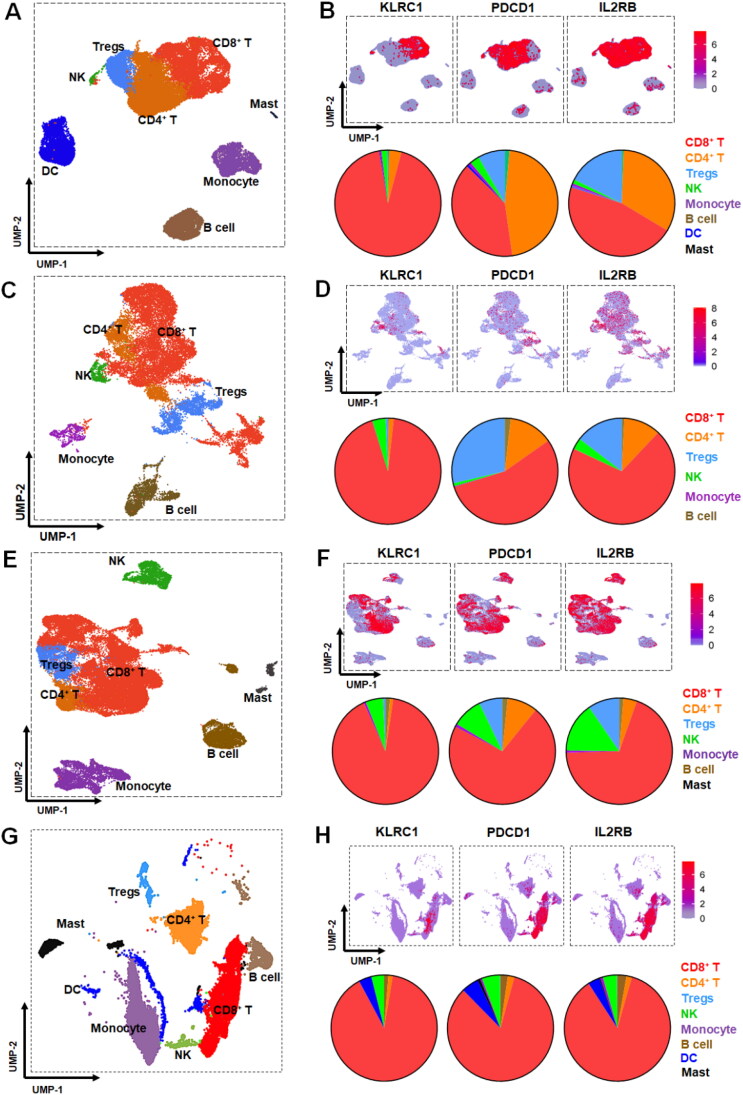
Expression profiles of NKG2A, PD-1, and IL2-Rβ tumor-infiltrated immune cells of cancer patients. (A, C, E, G) UMP plots showing main tumor-infiltrated immune cells of patients with Colon cancer (a), breast cancer (C), stomach cancer (E), or melanoma (G). (B, D, F, H) UMP plots and pie charts showing cells expressing NKG2A (KLRC1), PD-1 (PDCD1), and IL-2Rβ (IL2RB) in tumor-infiltrated CD45^+^ cells of patients with Colon cancer (B), breast cancer (D), stomach cancer (F), or melanoma (H). Single-cell transcriptome data were obtained from patients with Colon cancer (*N* = 23), breast cancer (*N* = 8), stomach cancer (*N* = 24), or melanoma (*N* = 12).

CD8^+^ T and NK cells are major antitumor immune effector cells (Geurts et al., 2023). Due to high expression of IL-2Rβγ in tumor-infiltrated CD8^+^ T and NK cells, delivery of an IL-2Rβγ agonist to these cells might enhance the intratumoral antitumor immune responses. However, IL-2Rβγ expression is not restricted to CD8^+^ T and NK cells, indicating a requirement for targeted delivery of the IL-2Rβγ agonist. To evaluate the potential of NKG2A as a target for the delivery of an IL-2Rβγ agonist to tumor-infiltrated CD8^+^ T and NK cells, the expression profile of NKG2A in immune cells derived from tumor tissues of cancer patients was analyzed and compared to that of PD-1, which is usually used as a target for the delivery of IL-2Rβγ agonist. As shown in Figure 1(A,B), the single-cell RNA sequencing of tumor tissues derived from 23 patients with colon cancer demonstrated that NKG2A was predominantly expressed in CD8^+^ T cells (93%), CD4^+^ T cells (3.7%), and NK cells (1.7%), whereas PD-1 was expressed in CD4^+^ T cells (46.3%), CD8^+^ T cells (39.5%), Tregs (8.2%), and NK cells (2.9%). In tumor tissues derived from 8 patients with breast cancer, NKG2A-expressing cells included CD8^+^ T cells (85.5%), NK cells (9.8%), and Tregs (3.8%). PD-1-expressing cells included CD8^+^ T cells (56.9%), Tregs (35.1%), NK cells (4.6%), B cells (2.3%), and dendritic cells (DC) (1%) (Figure 1(C,D)). In tumor-infiltrated immune cells derived from 24 patients with gastric cancer, 90.9%, 5.4%, and 0.7% of NKG2A-expressing cells were CD8^+^ T cells, NK cells, and Tregs, respectively, whereas the top PD-1-expressing cells were CD8^+^ T cells (71.8%), NK cells (9.4%), B cells (9.4%), Tregs (7.1%) (Figure 1(E,F)). In tumor-infiltrated immune cells derived from 12 melanoma patients, the expression rate of NKG2A in CD8^+^ T cells, NK cells, DC, B cells were 89.6%, 3.9%, 3.9%, and 1.3%, respectively, compared to 83.3%, 5.54%, 5.3%, and 2.3% of PD-1 in these cells (Figure 1(G,H)). These results demonstrated that, compared to PD-1, NKG2A exhibited a relatively narrow expression profile in human tumor-infiltrated immune cells.

In terms of the expression of NKG2A in immune effector cells, Figure 1(B,D,F,H) showed that the frequency of NKG2A (1.8% in colon cancer, 9.8% in breast cancer, 5.4% in stomach cancer, 3.9% in melanoma) in tumor-infiltrated NK cells was similar to that of PD-1 (2.9% in colorectal cancer, 4.6% in breast cancer, 9.4% in stomach cancer, and 5.5% in melanoma), whereas the frequency of NKG2A (93% in colorectal cancer, 85.5% in breast cancer, and 90.9% in stomach cancer, 89.6%) in tumor-infiltrated CD8^+^ T cells was higher than that of PD-1 (39.5% in colorectal cancer, 56.9% in breast cancer, 71.8% in stomach cancer, and 83.3% in melanoma). As IL-2Rβγ was predominantly expressed in tumor-infiltrated CD8^+^ T and NK cells (Figure 1(B,D,F,H)), these results indicated that, as a target candidate, NKG2A might be more efficient than PD-1 for delivery of IL-2Rβγ agonist to tumor-infiltrated immune effector cells. Notably, in addition to CD8^+^ T and NK cells, many Tregs were also IL-2Rβγ-expressive (Figure 1(B,D,F,H)). The frequency of NKG2A (0.2% in colorectal cancer, 3.8% in breast cancer, 0.7% in stomach cancer, and 0% in melanoma) in tumor-infiltrated Tregs was lower than that of PD-1 (8.2% in colorectal cancer, 35.1% in breast cancer, and 7.1% in stomach cancer, 0.14% in melanoma). Moreover, Figure S1 demonstrated that in tumor-infiltrated immune cells of colon cancer, breast cancer, gastric cancer, and melanoma, NKG2A and IL-2Rβ were predominantly co-expressed in CD8^+^ T cells (85–96.6%) and NK cells (1.7–11.5%). The co-localization rate of PD-1 with IL-2Rβ in CD8^+^ T cells (38.9–67.6%) was significantly lower than that of NKG2A with IL-2Rβ. In addition, the co-localization rate of PD-1 with IL-2Rβ in Tregs (6.4–43.8%) was significantly higher than that (0.3–5.6%) of NKG2A with IL-2Rβ. As IL-2Rγ usually co-exists with IL-2Rβ in immune cells, these results suggested that NKG2A with relative narrow expression profile might serve as a novel target for the delivery of IL-2Rβγ agonist, which might carry a lower risk to induce Tregs-mediated immunosuppression for human tumor immunotherapy.

### NKG2A and IL-2Rβγ are highly co-expressed in tumor-infiltrated CD8^+^ T and NK cells of mice

**Figure 2.**
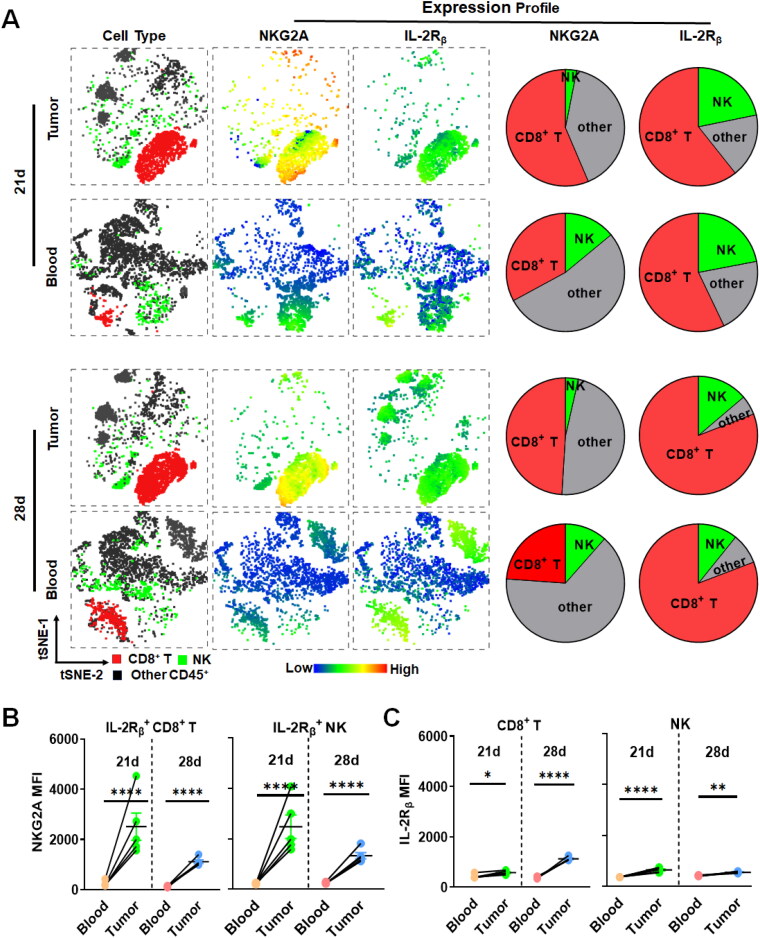
Expression profiles of NKG2A and IL2Rβ in peripheral and tumor-infiltrated immune cells of mice bearing MC38 tumor grafts. (A) tSNE and pie chart indicating NKG2A^+^, and IL-2Rβ^+^ cells in the main CD45^+^ cells from the blood and tumors. (B) Expression level of NKG2A in IL-2Rβ^+^ CD8^+^ T and IL-2Rβ^+^ NK cells. (C) Expression level of IL-2Rβ in CD8^+^ T and NK cells. Data are represented as mean ± SEM. *p < 0.05, **p < 0.01, ***p < 0.001, ****p < 0.0001. tSNE dimensionality reduction analysis of CD45^+^ immune subpopulations was performed in mice (*N* = 5) bearing MC38 tumor grafts.

To evaluate the potential of NKG2A as a target for delivery of IL2-Rβγ agonist in mice, expression profiles of NKG2A and IL2-Rβγ in tumor-infiltrated immune cells of mice bearing MC38 or B16/F1 tumor grafts were analyzed. As shown in Figure 2(A), tSNE plots indicated obvious distribution of NKG2A and IL-2Rβ in CD8^+^ T and NK cells in MC38 tumor tissues collected on 21d and 28d postinoculation. Of the NKG2A-expressing immune cells in tumor tissues collected on 21d, the percentage of CD8^+^ T and NK cells was 56.3% and 3.1%, respectively. Moreover, CD8^+^ T and NK cells accounted for 60.7% and 21.8% of the total IL-2Rβ-expressing immune cells in these tumor tissues, respectively (Figure 2(A)). Notably, as shown in Figure S2, NKG2A^+^IL2Rβγ^+^CD8^+^ T cells and NKG2A^+^IL2Rβγ^+^ NK cells accounted for 56–73.9% and 18.9–32.8% of NKG2A- and/or IL-2Rβγ-expressing tumor-infiltrated immune cells, indicating that NKG2A and IL2Rβγ were predominantly co-expressed in tumor-infiltrated CD8^+^ T and NK cells. Similar expression profiles of NKG2A and IL-2Rβγ were observed in CD8^+^ T and NK cells derived from MC38 tumor tissues collected on 28d. These results indicated that NKG2A and IL-2Rβγ were highly co-expressed in immune effector cells in MC38 tumor tissues. Moreover, in B16/F1 tumor tissues collected on 17d and 19d postinoculation, NKG2A-expressing CD8^+^ T and NK cells accounted for 51–56% of NKG2A-expressing immune cells, and IL-2Rβγ-expressing CD8^+^ T and NK cells accounted for 67–75% of IL-2Rβγ-expressing immune cells (Figure S3(A)), while NKG2A^+^IL2Rβγ^+^ CD8^+^ T cells and NKG2A^+^IL2Rβγ^+^ NK cells accounted for 38.6–69% and 18% of NKG2A- and/or IL-2Rβγ-expressing tumor-infiltrated immune cells (Figure S4), indicating high co-expression of NKG2A and IL-2Rβγ in CD8^+^ T and NK cells in B16/F1 tumor tissues. These results demonstrated that the expression profiles of NKG2A and IL-2Rβγ in tumor-infiltrated CD8^+^ T and NK cells of mice were similar to that of NKG2A and IL-2Rβγ in intratumoral CD8^+^ T and NK cells of humans, suggesting the feasibility and translational significance of orchestrating CD8^+^ T and NK cells by NKG2A-targeted delivery of IL-2Rβγ agonist for tumor immunotherapy in mice.

As intravenously administrated NKG2A-targeted IL-2Rβγ agonist might be captured by NKG2A- and/or IL-2βγ-expressing cells in blood, which would reduce the tumor uptake of NKG2A-targeted IL-2Rβγ agonist, the expression profiles of NKG2A and IL-2βγ in PBMCs of mice bearing MC38 (Figure 2) or B16/F1 (Figure S3) tumor grafts were also examined and compared to those of NKG2A and IL-2βγ in tumor-infiltrated immune cells. As shown in Figures 2(A) and S3(A), in addition to being expressed in CD8^+^ T (23–33%) and NK cells (11.5–21%), NKG2A was also expressed in other PBMCs (41–69%), suggesting that the intravenously administrated NKG2A-targeted IL-2Rβγ agonist might be predominantly captured by these cells in blood, which might reduce the tumor uptake of the NKG2A-targeted IL-2Rβγ agonist. However, further analysis demonstrated that the levels of both NKG2A (Figure 2(B)) and IL-2Rβγ (Figure 2(C)) in tumor-infiltrated CD8^+^ T (MFI 1108–2560) and NK cells (MFI 1331–2484) were significantly higher than those of NKG2A and IL-2Rβγ in peripheral CD8^+^ T (MFI 124–230) and NK cells (MFI 222–244) derived from mice bearing MC38 tumor grafts. Moreover, in mice bearing B16/F1 tumor grafts, although the expression of IL-2Rβγ in tumor-infiltrated CD8^+^ T (MFI 208–250) and NK cells (MFI 291–430) was comparable to that of IL-2Rβγ in peripheral CD8^+^ T (MFI 172–223) and NK cells (MFI 240–373) (Figure S3(C)), the levels of NKG2A in tumor-infiltrated CD8^+^ T (MFI 947–1352) and NK cells (MFI 899–2036) were also significantly higher than those of NKG2A in peripheral blood CD8^+^ T (MFI 83–376) and NK cells (MFI 377–459) (Figure S3(B,C)). The increased expression of NKG2A and IL-2Rβγ in tumor-infiltrated immune effector cells suggested that the majority of intravenously administrated NKG2A-targeted IL-2Rβγ agonist would be delivered into tumor grafts of mice.

### IL-2βγ agonist N215 could be coupled to immune checkpoint inhibitor αNKG2A to produce αNKG2A-N215 with dual specificities

**Figure 3.**
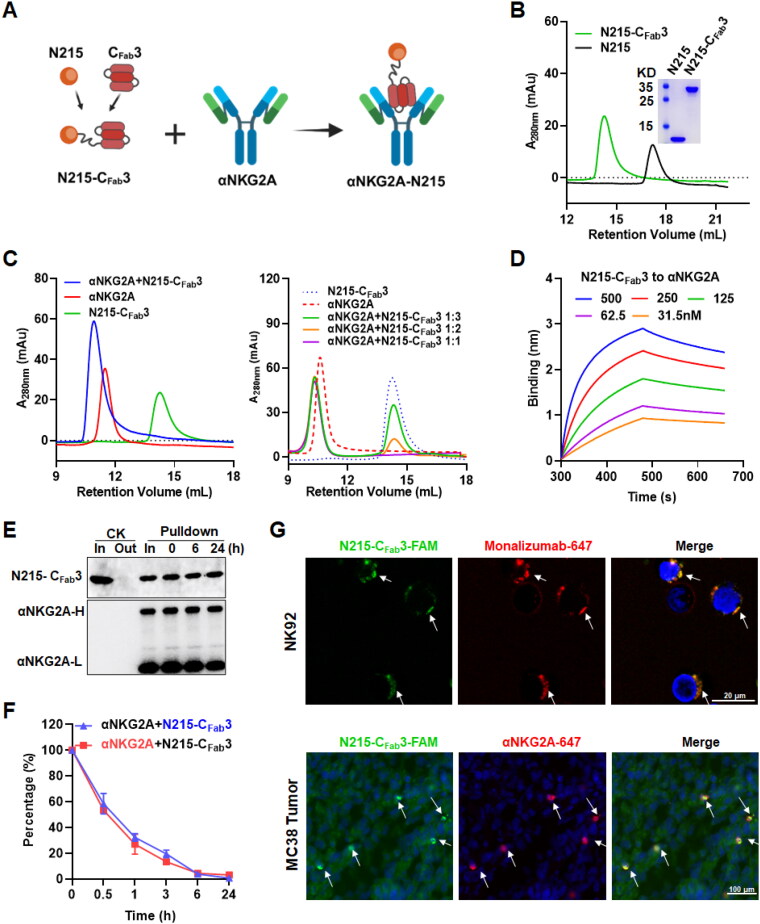
Coupling of N215 to IgG antibody against murine NKG2A. (A) Schematic diagram for Fab-binding domain-mediated coupling of N215 to αNKG2A. (B) SDS-PAGE and SEC analysis of purified N215 with (N215-C_Fab_3) or without (N215) IgBD. (C) Binding of N215-C_Fab_3 to αNKG2A analyzed by SEC. (D) Affinity of N215-C_Fab_3 for αNKG2A measured by biolayer interferometry. (E) Pulldown of αNKG2A bound to N215-C_Fab_3 in mouse serum. (F) Blood clearance of αNKG2A and N215-C_Fab_3 in the format of complex. After intravenous injection of the complexes into mice (*N* = 3), the residual αNKG2A and N215-C_Fab_3 in the blood were measured by ELISA, respectively. (G) Co-localization of αNKG2A and N215-C_Fab_3 on cells under *in vitro* as well as *in vivo* conditions. Antibody (αNKG2A and manolizumab) and N215-C_Fab_3 were labeled with alexa fluor 647 or FAM for preparation of complexes. The complexes of N215-C_Fab_3 and manolizumab were incubated with NK92 cells prior to observation. The complexes of N215-C_Fab_3 and αNKG2A were intravenously injected into mice bearing MC38 tumor grafts followed by collection at 4 h postinjection for observation. The nuclei were visualized by DAPI staining.

To prepare NKG2A-targeted IL-2Rβγ agonist by coupling a single N215 molecule to an IgG antibody against NKG2A, C_Fab_3 was fused to N215 to produce N215-C_Fab_3 (Figure 3(A)). As shown in Figure 3(B), N215-C_Fab_3 recovered from the supernatant of *E. coli* was visualized as a single protein band on SDS-PAGE gel and a single protein peak on SEC column with the predicted molecular weight, indicating that N215-C_Fab_3 was purified to homogeneity. Similarly, N215 without the C_Fab_3 was also purified to homogeneity in the same way. To determine the binding of N215-C_Fab_3 to αNKG2A against murine NKG2A, N215-C_Fab_3 was mixed with αNKG2A at an equal molar ratio followed by separation on SEC column. As shown in Figure 3(C), a novel protein peak with a molecular weight that was larger than that of αNKG2A was observed in the mixture of αNKG2A and N215-C_Fab_3 (αNKG2A + N215-C_Fab_3), whereas the mixture of αNKG2A and N215 (αNKG2A + N215) was separated as two protein peaks corresponding to individual αNKG2A and N215 by SEC (Figure S5(A)), indicating that N215-C_Fab_3 containing IgBD could bind αNKG2A. Moreover, when αNKG2A was mixed with N215-C_Fab_3 at a molar ratio of 1:1, a single protein peak was visualized in the mixture by SEC. Nevertheless, excessive N215-C_Fab_3 was measured in the mixture when αNKG2A was mixed with N215-C_Fab_3 at a molar ratio of 1:2 or 1:3 (Figure 3(C)), suggesting that N215-C_Fab_3 bound to αNKG2A at a ratio of 1:1. Subsequent protein-protein interaction assays verified the binding of N215-C_Fab_3 to αNKG2A with high affinity (KD = 19.8 nM) (Figure 3(D)). To determine whether the bound αNKG2A would be disassociated from N215-C_Fab_3 by endogenous IgG, the complexes of αNKG2A and N215-C_Fab_3 were added into mouse serum prior to the pulldown experiment. Figure 3(E) demonstrated that N215-C_Fab_3 was consistent with αNKG2A in the pulldown products, indicating that the bound αNKG2A was not disassociated from N215-C_Fab_3 by murine IgGs in serum under *in vitro* conditions. To further determine whether the αNKG2A would be detached from N215-C_Fab_3 under *in vivo* conditions, the complexes of αNKG2A and N215-C_Fab_3 were intravenously injected into mice followed by measuring residual αNKG2A or N215-C_Fab_3 in the blood collected at different time postinjection. As shown in Figure 3(F), the blood clearance rates of αNKG2A and N215-C_Fab_3 within 24 h were highly similar, suggesting that premixed αNKG2A and N215-C_Fab_3 existed as a complex in the blood. Figure 3(G) demonstrated that αNKG2A was well co-localized with N215-C_Fab_3 on cells under *in vitro* as well as *in vivo* conditions. These results demonstrated that, under the mediation by IgG-binding C_Fab_3, a single N215 molecule could be coupled to an αNKG2A antibody to form a stable complex, i.e. αNKG2A-N215. Similarly, protein-protein interaction assays demonstrated that N215-C_Fab_3 bound human antibody against human NKG2A, i.e. Monalizumab, with high affinity (KD = 95.3 nM) (Figure S5(B)). Further pulldown experiments demonstrated that the bound Monalizumab was not disassociated from N215-C_Fab_3 by other human IgGs in serum under *in vitro* conditions (Figure S5(C)), suggesting that Monalizumab and N215-C_Fab_3 also formed a stable complex that was designated as Monalizumab-N215.

**Figure 4.**
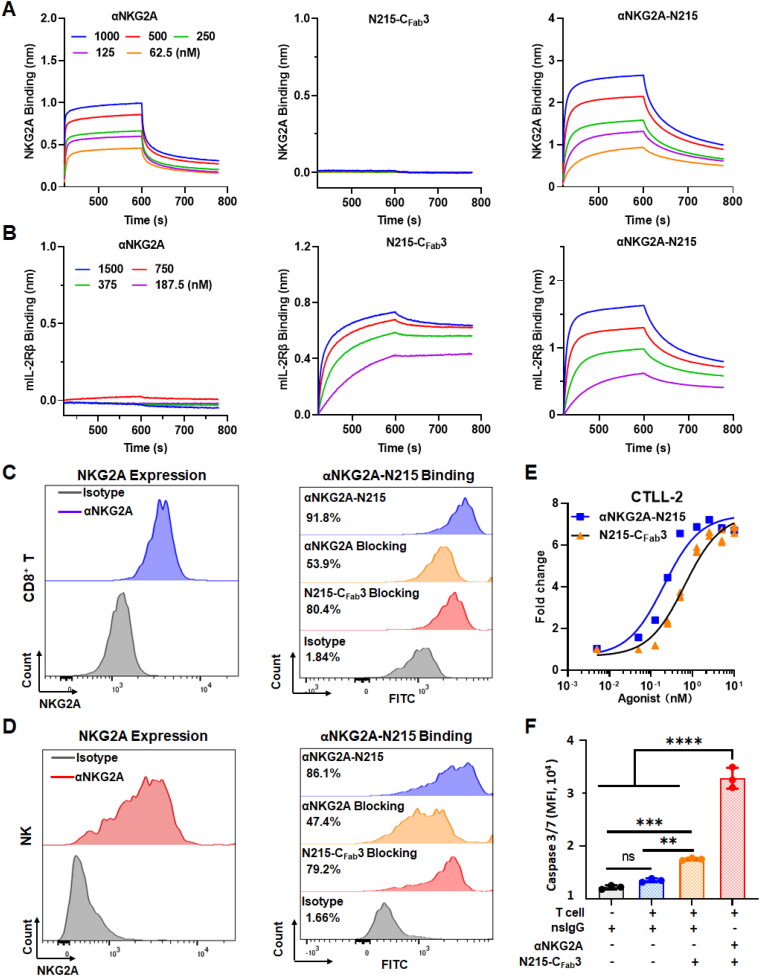
Dual specificities of αNKG2A-N215 for NKG2A and IL-2Rβ. (A, B) Affinity of αNKG2A-N215, αNKG2A, and N215-C_Fab_3 for murine NKG2A (A) and IL-2 Rβ (B) measured by biolayer interferometry. (C, D) Binding of αNKG2A-N215 to murine CD8^+^ T cells (C) and NK cells (D). (E) Proliferation of CTLL-2 stimulated by αNKG2A-N215 or N215-C_Fab_3. (F) Tumor cell death induced by T cells in the presence of proteins. Data are represented as mean ± SEM. *p < 0.05, **p < 0.01, ***p < 0.001, ****p < 0.0001, ns: not significant.

Protein-protein interaction assays demonstrated that the affinity of αNKG2A-N215 for murine NKG2A was comparable to that of αNKG2A (Figure 4(A)). Moreover, αNKG2A-N215 and N215-C_Fab_3 showed similar binding to murine IL-2Rβ (Figure 4(B)). Accordingly, Monalizumab-N215 and N215-C_Fab_3 showed similar binding to human IL-2Rβγ (Figure S6(A)). These results demonstrated that the NKG2A-binding of αNKG2A and IL-2Rβ-binding of N215-C_Fab_3 were well preserved in αNKG2A-N215. Cell-binding assays demonstrated that αNKG2A-N215 could bind murine CD8^+^ T cells and NK cells (Figures 4(C,D) and S7). The binding rate of αNKG2A-N215 to CD8^+^ T cells was decreased from 91.8% to 53.9% by blocking with αNKG2A or decreased from 91.8% to 80.4% by blocking with N215-C_Fab_3 (Figure 4(C)). Similarly, the binding rate of αNKG2A-N215 to NK cells was also decreased from 86.1% to 47.4% by blocking with αNKG2A or decreased from 86.1% to 79.2% by blocking with N215-C_Fab_3 (Figure 4(D)). These results indicated that the binding ability of αNKG2A to NKG2A and that of N215-C_Fab_3 to IL-2Rβγ were preserved in αNKG2A-N215. Similar to N215-C_Fab_3, αNKG2A-N215 also effectively stimulated *in vitro* proliferation of T cells (Figure 4(E)) and NK cells (Figure S6(B)). In addition, αNKG2A-N215 enhanced the killing of tumor cells by T cells (Figure 4(F)). These results indicated that αNKG2A-N215 exhibited dual specificities for NKG2A and IL-2Rβγ, which were essential to orchestrating both CD8^+^ T and NK cells for tumor immunotherapy.

### αNKG2A-N215 treatment induces greater antitumor immune responses predominantly by orchestrating intratumoral CD8^+^ T and NK cells in mice

**Figure 5.**
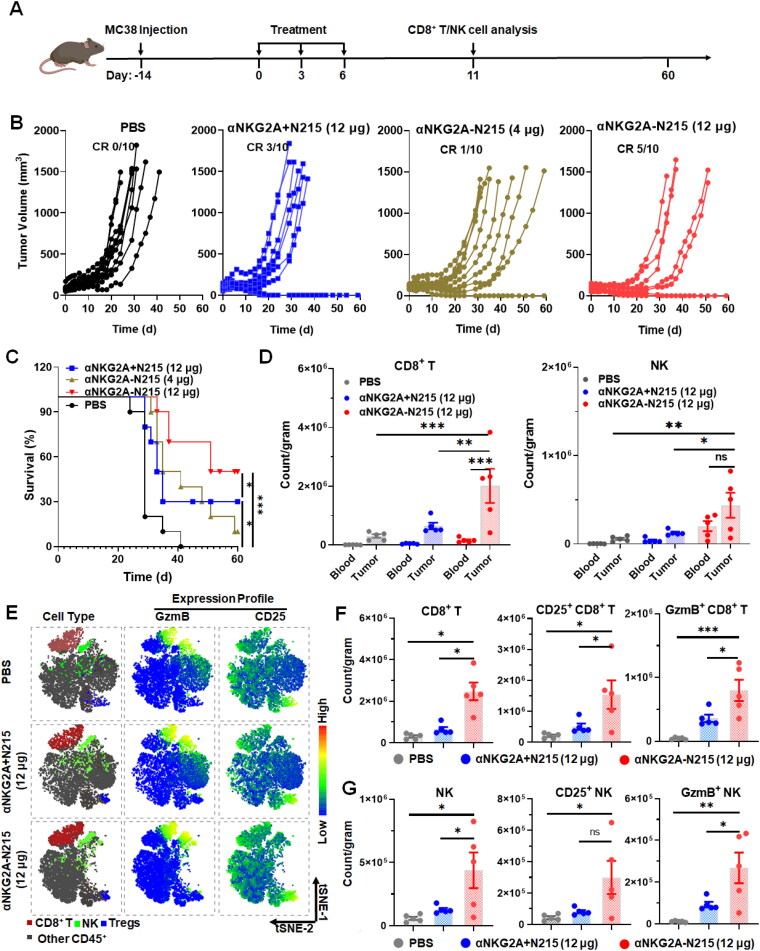
Antitumor effect of αNKG2A-N215 in mice bearing MC38 tumor grafts. (A) Schedule for treatment and analysis. (B) Perspective growth curves of tumor grafts after treatment with αNKG2A-N215 or combination therapy of αNKG2A and N215. Proteins were intravenously injected into mice (*N* = 10) bearing MC38 tumor grafts every 3 days for a total of 3 injections. (C) Survival rates of treated mice. (D) CD8^+^ T and NK cells in the blood or tumors at 11d after initial treatment. (E) tSNE plots indicating GzmB-, CD25-expressing cells in tumor-infiltrated immune cells. (F, G) Tumor-infiltrated CD8^+^ T (E) and NK (F) cells in mice (*N* = 5). Data are represented as mean ± SEM. *p < 0.05, **p < 0.01, ***p < 0.001, ns: not significant.

The antitumor effect of αNKG2A-N215 was evaluated in mice bearing subcutaneous MC38 or B16/F1 tumor grafts and compared to that of the separate combination of αNKG2A and N215 (αNKG2A + N215) (Figure 5(A)). As shown in Figure 5(B), although 3 out of 10 mice bearing MC38 tumors showed complete response to the mixture of αNKG2A and N215, administration of αNKG2A + N215 (12 μg) did not obviously suppress the growth of tumors in remaining mice. However, treatment with αNKG2A-N215 at a dose of 4 μg N215 equivalent (αNKG2A-N215 (4 μg)) obviously retarded the tumor growth. When the dose of αNKG2A-N215 was increased to 12 μg N215 equivalent (αNKG2A-N215 (12 μg)), tumor grafts in 5 out of 10 mice were eradicated after treatment for 2 weeks. Figure 5(C) showed that the median survival time for mice treated with αNKG2A-N215 (12 μg) was 55.5 days, which was significantly longer than that (34 days) of mice treated with αNKG2A + N215 (12 μg), indicating that αNKG2A-N215 treatment was superior to the separate combination therapy of αNKG2A and N215 in increasing the survival rate as well as extending the survival time of mice bearing MC38 tumor grafts.

Immune effector cells assays demonstrated that the number of tumor-infiltrated CD8^+^ T and NK cells in mice treated with αNKG2A-N215 (12 μg) was 3–4 times greater than that in mice treated with αNKG2A + N215 (12 μg) (Figure 5(D)). Functional indicator analysis demonstrated that CD25-expressing (CD25^+^) and granzyme B-expressing (GzmB^+^) CD8^+^ T and NK cells in tumor grafts treated with αNKG2A-N215 (12 μg) were also 2–4 times more than those in tumor grafts treated with αNKG2A + N215 (12 μg) (Figures 5(E,F,G) and S8). However, the number of tumor-infiltrated Tregs in mice treated with αNKG2A-N215 (12 μg) was not significantly higher than that in mice treated with αNKG2A + N215 (12 μg). Consequently, the ratio of CD8^+^ T cells to Tregs in tumor grafts treated with αNKG2A-N215 (12 μg) was significantly (*P* < 0.05) higher than that in tumor grafts treated with αNKG2A + N215 (Figure S9), which was consistent with the difference between these two treatments in their antitumor effect. Notably, although the trend of immune effector cell changes in the peripheral blood (Figure S10) was consistent with that in the tumor (Figure 5(D)). In mice treated with αNKG2A-N215, tumor-infiltrated CD8^+^ T and NK cells were 12.6 folds and 2.7 folds those of peripheral immune effector cells (Figure 5(D)), respectively, indicating that αNKG2A-N215 predominantly induced intratumoral antitumor immune responses. Although the counts of CD8^+^ T cells (20.1 × 10^5^ cells/gram) were significantly (*P* < 0.01) greater than those of NK cells (4.4 × 10^5^ cells/gram) in tumor grafts treated with αNKG2A-N215 (Figure 5(E,F)), the increasing degree of NK cells (7.5 folds) was comparable to that of CD8^+^ T cells (8.1 folds), indicating that αNKG2A-N215 exerted antitumor effect by orchestrating both CD8^+^ T and NK cells in mice bearing MC38 tumor grafts.

**Figure 6.**
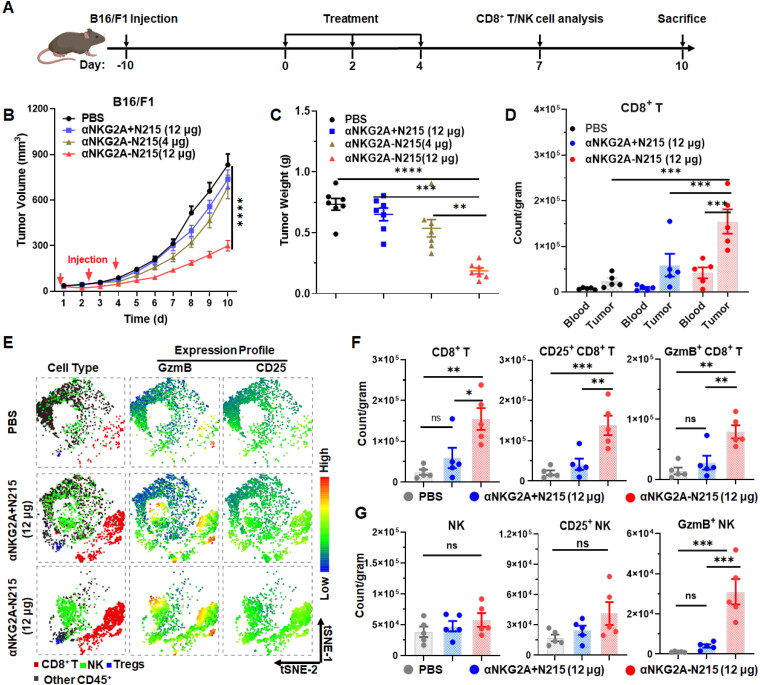
Antitumor effect of αNKG2A-N215 evaluated in mice bearing B16/F1 tumor grafts. (A) Schedule for treatment and analysis. (B)Growth curves of tumor grafts after treatment with αNKG2A-N215 or combination therapy of αNKG2A and N215. Mice (*N* = 7) were intravenously injected every 2 days for a total of 3 injections. (C) Tumor weight of treated mice. (D) CD8^+^ T and NK cells in blood and tumor tissue at 7d after initial treatment. (E) tSNE plots indicating GzmB-, and CD25-expressing cells in intratumoral immune cells of treated mice. (F, G) Tumor-infiltrated CD8^+^ T (F) and NK (G) cells at 7d after initial treatment. Data are represented as mean ± SEM. *p < 0.05, **p < 0.01, ***p < 0.001, ****p < 0.0001, ns: not significant.

In addition, antitumor effect of αNKG2A-N215 was also evaluated in mice bearing B16/F1 tumor grafts (Figure 6(A)). As shown in Figure 6(B), treatment with either αNKG2A + N215 (12 μg) or αNKG2A-N215 (4 μg) showed little suppression on tumor growth. However, administration of αNKG2A-N215 (12 μg) significantly (*P* < 0.0001) inhibited the growth of B16/F1 tumor grafts. At the end of observation, the mean tumor volume of mice treated with αNKG2A-N215 (12 μg) was 299.1 ± 34.3 mm^3^, compared to 736.6 ± 60.9 mm^3^ for mice treated with αNKG2A + N215 (12 μg). Accordingly, the mean tumor weight of mice treated with αNKG2A-N215 (12 μg) was significantly (0.2 ± 0.1 g vs 0.7 ± 0.1 g, *P* < 0.0001) lighter than that of mice treated with αNKG2A + N215 (12 μg) (Figures 6(C) and S11). These results demonstrated that αNKG2A-N215 treatment showed greater antitumor effect than the separate combination of αNKG2A and N215. Mechanistically, it was also found that αNKG2A-N215 treatment predominantly induced a greater number of CD8^+^ T and NK cells in tumor grafts while showing little impact on Tregs in mice bearing B16/F1 tumor grafts (Figures 6(D,E,F,G) and S12(A,B)). The ratio of CD8^+^ T cells to Tregs in tumor grafts treated with αNKG2A-N215 (12 μg) was significantly (*P* < 0.01) higher than that in tumor grafts treated with αNKG2A + N215 (Figure S12(C)), which was consistent with the difference between these two treatments in their antitumor effect. These results demonstrated that αNKG2A-N215 with dual specificities for NKG2A and IL-2Rβγ induced more robust antitumor immune responses by orchestrating both CD8^+^ T and NK cells. Moreover, the counts of CD8^+^ T and NK cells in tumors (Figure 6(D,F,G)) treated with αNKG2A-N215 (12 μg) were 3.7 times and 14.1 times those in blood (Figures S12(B) and S13), respectively, indicating that αNKG2A-N215 treatment predominantly induced intratumoral antitumor immune responses in mice bearing B16/F1 tumors.

### αNKG2A-N215 treatment induces long-term immunological memory against the tumor

**Figure 7.**
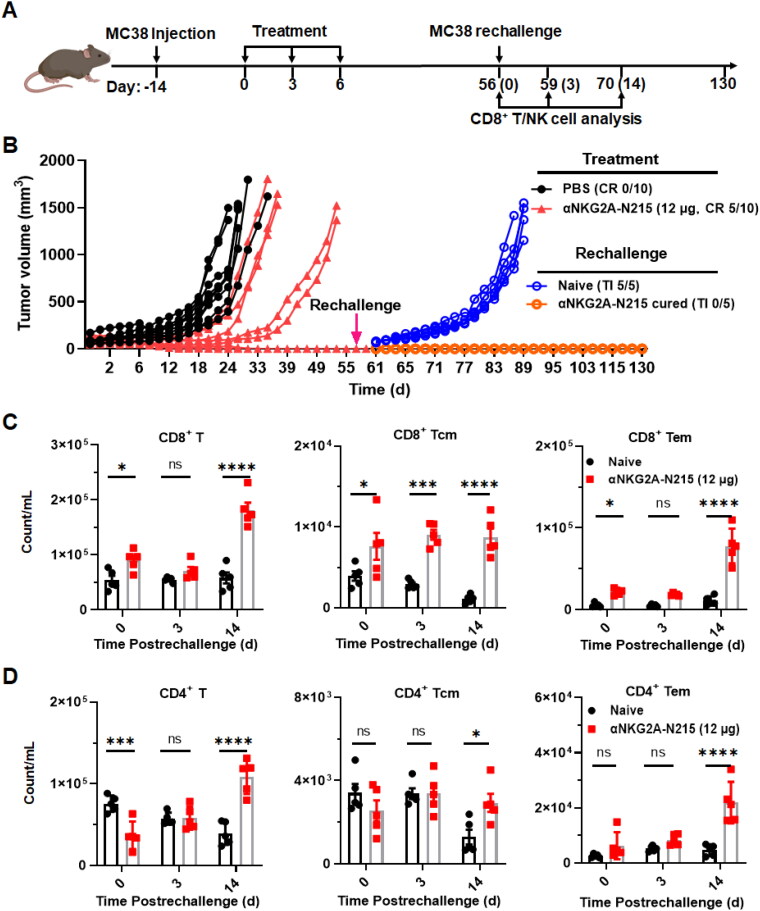
Long term immunological memory induced by αNKG2A-N215. (A) Schedule for rechallenge and analysis. (B) Rechallenge of αNKG2A-N215 cured mice (*N* = 5) with MC38 cells. αNKG2A-N215 cured mice described in Figure 5 were rechallenged with MC38 tumor cells followed by monitoring the tumor incidence (TI). Naïve mice (*N* = 5) were used as control. (C, D) CD8^+^ (C) and CD4+ (D) T memory cells in the blood of mice at different times after rechallenge. Data are represented as mean ± SEM. *p < 0.05, **p < 0.01, ***p < 0.001, ****p < 0.0001, ns: not significant.

In mice bearing MC38 tumor grafts, tumor eradication was observed in 5 out of 10 mice within 2 weeks after treatment with αNKG2A-N215 (12 μg) and reoccurrence did not happen in these mice after treatment for over 50 days. To investigate whether αNKG2A-N215 treatment induced long-term immunological memory against the tumor, these αNKG2A-N215-cured mice were rechallenged with MC38 tumor cells with naïve mice as a control (Figure 7(A)). As shown in Figure 7(B), after inoculation of MC38 tumor cells for one week, tumor incidence was observed in all naïve mice. In contrast, no tumor was observed in any of αNKG2A-N215-cured mice at the end of observation (over 70 days after rechallenge), suggesting that αNKG2A-N215 induced long-term immunological memory against the tumor. To verify these results, immunological memory-associated T cells including central memory T cells (CD44^high^CD62L^high^, Tcm) and effector/memory T cells (CD44^high^CD62L^low^, Tem) in the blood of rechallenged mice were measured and compared to those of mice before rechallenge. Figure 7(C,D) demonstrated that little change was observed in CD8^+^ Tcm and CD4^+^ Tcm within two weeks after rechallenge. However, both CD8^+^ Tem and CD4^+^ Tem cells were increased for 8.6 and 5.6 times (10.8 × 10^3^ vs 77.9 × 10^3^ cells/mL for CD8^+^ Tem, 4.7 × 10^3^ vs 21.9 × 10^3^ cells/mL for CD4^+^ Tem), respectively, after rechallenge of the mice for two weeks, indicating that αNKG2A-N215 treatment induced long-term immunological memory against tumor.

## Discussion

Administration of ICIs such as antibodies against PD-1 or NKG2A could partially normalize the effector functions of both T and NK cells by eliminating the brake effect of immune checkpoints. However, only a limited number of patients showed complete responses to monotherapy based on present ICIs (van Montfoort et al., 2018, Roe, 2022, Ducoin et al., 2022, Jia et al., 2023). In recent years, more and more studies have demonstrated that, during the progressive exhaustion of T and NK cells, increased expression of immune checkpoints is usually accompanied by decreased production of proliferative cytokine, such as IL-2 (Kyrysyuk and Wucherpfennig, 2023), suggesting that combination therapy of ICIs and IL-2 might induce greater antitumor immune responses. As PD-1 is the most extensively studied immune checkpoints, several fusion proteins containing antibodies against PD-1 and IL-2 variant have been produced for tumor immunotherapy, which have exhibited significantly improved antitumor effects in preclinical tests (Piper et al., 2023, Tichet et al., 2023).

Notably, as both PD-1 and IL-2R are also expressed on other immune cells including immunosuppressive Tregs (Zhong et al., 2015), PD-1-targeted delivery of an IL-2 variant might also stimulate proliferation of Tregs, indicating the need to identify another target. According to data of patients with different cancers, the expression profile of NKG2A was narrower than that of PD-1 in tumor-infiltrated immune cells. NKG2A was predominantly co-expressed with IL-2Rβγ in CD8^+^ T and NK cells (Figure 1). In contrast, co-expression of PD-1 and IL-2Rβγ was also observed in a few Tregs and other CD4^+^ T cells (Figure S1), suggesting that NKG2A-targeted delivery of an IL-2 variant might induce lower off-target-associated side effects. Especially, the expression profiles of NKG2A and IL-2Rβγ in tumor-infiltrated immune cells of mice (Figures 2 and S1) were highly similar to those in humans, indicating high reliability and clinical translation of NKG2A-targeted delivery of an IL-2 variant evaluated in mice. These results greatly triggered our interest in developing NKG2A-targeted IL-2 variant for tumor immunotherapy. To develop an NKG2A-targeted IL-2 variant, evidently, an off-the-shelf therapeutic antibody against NKG2A is an ideal carrier molecule. For antibody selection, due to the difference in specificities, antibodies against murine (αNKG2A) or human (Monalizumab) NKG2A were used to develop NKG2A-targeted IL-2 variant. For IL-2 variant selection, due to the overexpression of IL-2Rα in Tregs, variants with reduced IL-2Rα affinity produced by site-directed mutation of native IL-2 were used in previous studies (Siebert et al., 2022, Piper et al., 2023, Tichet et al., 2023). Nevertheless, strategies for IL-2 variant production by natural protein evolution could not completely abrogate IL-2Rα-binding while preserving strong IL-2Rβγ-binding (Quijano-Rubio et al., 2020). Interestingly, an IL-2/IL-15 mimic, i.e. N215, was produced by state-of-the-art de novo computational and experimental protein design recently (Silva et al., 2019), which fully eliminated the binding site and dependency on IL-2Rα while enhancing the agonistic activity toward IL-2Rβγ, suggesting that N215 was valuable as a payload for NKG2A-targeted delivery for tumor immunotherapy.

Theoretically, N215 could be fused or coupled to antibody against NKG2A. In previous studies, IL-2 variants were usually genetically fused to antibody (Klein et al., 2017, Siebert et al., 2022, Piper et al., 2023, Tichet et al., 2023). As antibodies are bivalent, conventional genetic fusion would incorporate at least two cytokine molecules to a single antibody molecule. Due to the super activity of cytokines, only a low dose of fusion protein could be administrated. Under this circumstance, the amount of antibody in the fusion protein might be insufficient. Fuzing one cytokine molecule to the antibody by using the knob-into-holes technology could reduce the amount of cytokine, thereby increasing the amount of antibody (Klein et al., 2017), which is especially important for immune checkpoint-targeted delivery of cytokines. However, difficulty of the knob-into-holes technology makes IgBD-mediated coupling an attractive way to produce engineered antibody. In our previous study, it was found that the trivalent, but not the monovalent IgBD exerted high affinity for IgGs (Fan et al., 2021). Consequently, in this paper, C_Fab_3 containing three tandemly repeated IgBD was fused to N215 to endow N215 with IgG-binding ability. It was found that N215-C_Fab_3 bound αNKG2A at a ratio of 1:1 (Figure 3(C)), allowing us to couple a single N215 molecule to each antibody. The high affinity of N215-C_Fab_3 for IgG antibodies, including αNKG2A and Monalizumab, prevented their disassociation in the presence of competitive IgGs (Figures 3(D,E) and S5(B,C)). After binding, N215-C_Fab_3 and αNKG2A showed similar blood clearance (Figure 3(F)) and cellular distribution in tumor (Figure 3(G)), indicating that N215-C_Fab_3 and αNKG2A formed a stable complex that was designated as αNKG2A-N215. Moreover, protein-protein interaction assays demonstrated that αNKG2A-N215 showed dual specificities for NKG2A (Figure 4(A)) and IL-2Rβγ (Figure 4(B)), indicating that N215 could be easily coupled to αNKG2A by fusion to the affinity ligand C_Fab_3.

*In vitro* assays demonstrated that αNKG2A-N215 could not only stimulate proliferation (Figure 4(E) and S6(B)) but also enhance cytotoxicity of immune cells (Figure 4(F)). Treatment of mice bearing MC38 (Figure 5(B,C)) or B16/F1 (Figure 6(B,C)) tumor grafts with αNKG2A-N215 showed greater antitumor effect than that achieved by the separate combination therapy of αNKG2A and N215. Accordingly, tumor-infiltrated CD8^+^ T and NK cells, especially those with effector phenotypes (granzyme B and CD25) in mice treated with αNKG2A-N215 were significantly more numerous than those in mice treated with the combination therapy of αNKG2A and N215 (Figures 5 and 6). These results indicate that coupling of N215 to αNKG2A is more efficient than the separate combination of N215 and αNKG2A to induce antitumor immune responses. After treatment with αNKG2A-N215, both tumor-infiltrated granzyme B- and CD25-expressing CD8^+^ T and NK cells were drastically increased, indicating that αNKG2A-N215 orchestrated both CD8^+^ T and NK cells to combat tumors. Notably, CD8^+^ T and NK cells in tumor were significantly more numerous than those in blood (Figures 5(D), 6(D), and S12(B)), indicating that αNKG2A-N215 treatment predominantly induced intratumoral antitumor immune responses, which was consistent with the higher expression of NKG2A in tumor-infiltrated immune effector cells (Figures 2 and S3). Similarly, increased NKG2A expression in tumor-infiltrated immune effector cells was also reported in many human cancers (Sun et al., 2017, van Montfoort et al., 2018, Abd Hamid et al., 2019), suggesting that αNKG2A-N215 treatment might also predominantly induce intratumoral immune responses. Attractively, αNKG2A-N215 treatment also induced memory T cells in some mice (Figure 7), indicating that it is valuable to evaluate the potential of αNKG2A-N215 for tumor reoccurrence control in future.

Taken together, our results demonstrated that αNKG2A-N215 may orchestrate both T and NK cells by using αNKG2A to brake off the immune check of NKG2A as well as by using N215 to trigger IL2-Rβγ, which reversed the tumor-induced T and NK cell exhaustion thus enhanced the antitumor immune responses. Especially, eradication of tumor grafts in some mice administrated with αNKG2A-N215 was accompanied by production of memory T cells, indicating that antitumor T and NK cells orchestrated by αNKG2A-N215 induced long-term immune memory against tumor cells, which might prevent patients from tumor reoccurrence. These results indicate the translational potential of αNKG2A-N215 in clinical cancer treatment.

## Conclusions

NKG2A is predominantly co-expressed with IL-2Rβγ in both human and murine CD8^+^ T and NK cells, indicating its potential as a novel target for delivery of IL-2Rβγ agonists for tumor immunotherapy. An NKG2A-targeted IL-2Rβγ agonist could be easily produced by coupling N215 containing IgBD to an off-the-shelf antibody against NKG2A. The novel NKG2A-targeted IL-2Rβγ agonist induces robust antitumor immune responses by orchestrating both T and NK cells. These results demonstrate that the NKG2A-targeted IL-2Rβγ agonist is valuable for further evaluation for tumor immunotherapy.

## Supplementary Material

Supplemental Material

## Data Availability

All data are available from the corresponding author on reasonable request.
