# Supplementary material for: Orchestrating T and NK cells for tumor immunotherapy via NKG2A-targeted delivery of a de novo designed IL-2Rβγ agonist
Source: Drug Deliv. 2025 Apr 1;32(1):2482195. doi: 10.1080/10717544.2025.2482195 (PMC11966987; doi:10.1080/10717544.2025.2482195)
Supplement: Supplemental Material [file IDRD_A_2482195_SM2480.docx]

**Supplementary materials**

**Orchestrating T and NK cells for tumor immunotherapy via NKG2A-targeted delivery of a de novo designed IL-2Rβγ agonist**

Jie Chen^1^, Enhui Ren^1^, Ze Tao^1,2,3^, Hongyu Lu^1^, Yunchuan Huang^1^, Jing Li^1^, Yuzhe Chen^1^, Zhuo Chen^1^, Tianshan She^1^, Hao Yang^1,2,3^, Hong Zhu^4#^, Xiaofeng Lu^1,2,3#^

1 Division of Abdominal Tumor Multimodality Treatment, Cancer Center; NHC Key Lab of Transplant Engineering and Immunology, Regenerative Medicine Research Center, West China Hospital, Sichuan University, Chengdu 610041, China

2 Sichuan Provincial Engineering Laboratory of Pathology in Clinical Application, West China Hospital, Sichuan University, Chengdu 610041, China

3 Institutes for Systems Genetics, Frontiers Science Center for Disease-Related Molecular Network, West China Hospital, Sichuan University, Chengdu 610041, China

4 Division of Abdominal Tumor Multimodality Treatment, Cancer Center, West China Hospital, Sichuan University, Chengdu 610041, China

#Corresponding author, Xiaofeng Lu, E-mail: xiaofenglu@scu.edu.cn; Hong Zhu, E-mail: zhuhong938@wchscu.cn


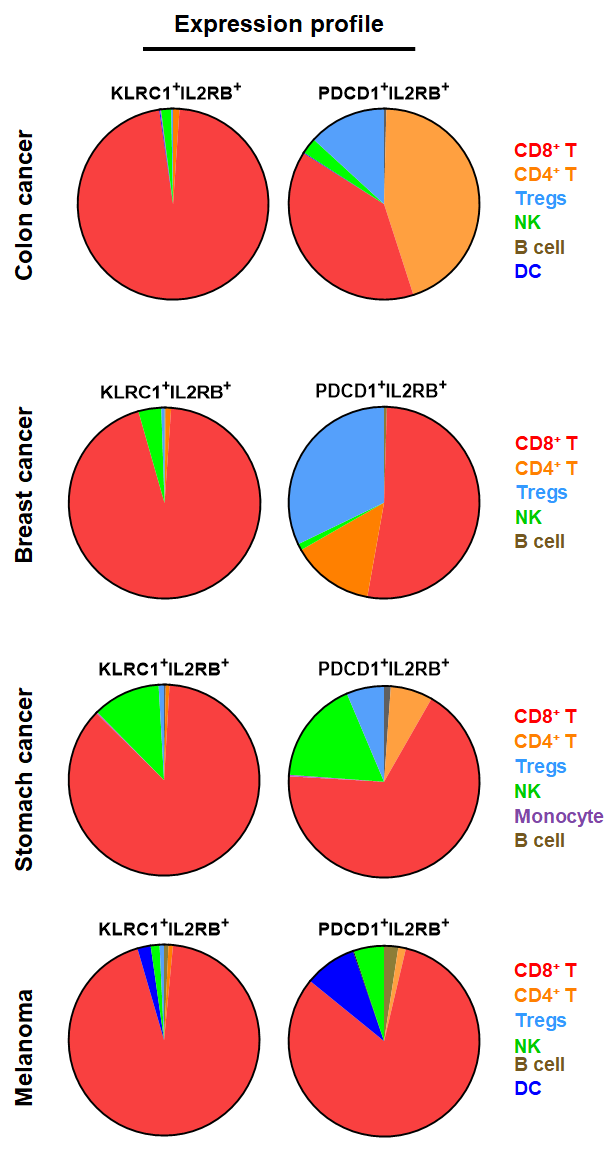


**Fig. S1 Co-expression of NKG2A (KLRC1) and IL-2Rβ (IL-2RB) in tumor-infiltrated immune cells of cancer patients.**

**
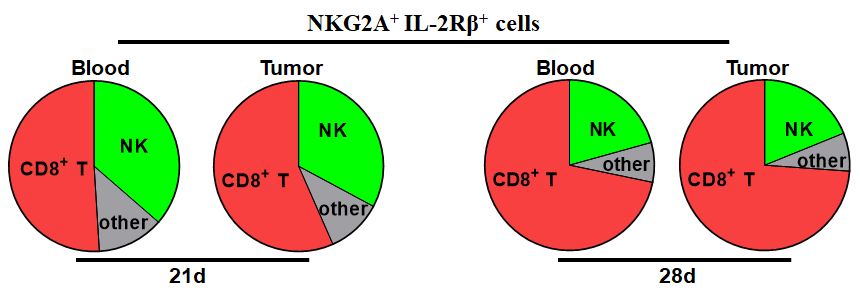
**

**Fig. S2 Pie charts indicating IL-2Rβ^+^NKG2A^+^ cells in the main CD45^+^ cells from the blood and tumors of mice bearing MC38 tumor grafts.**

**
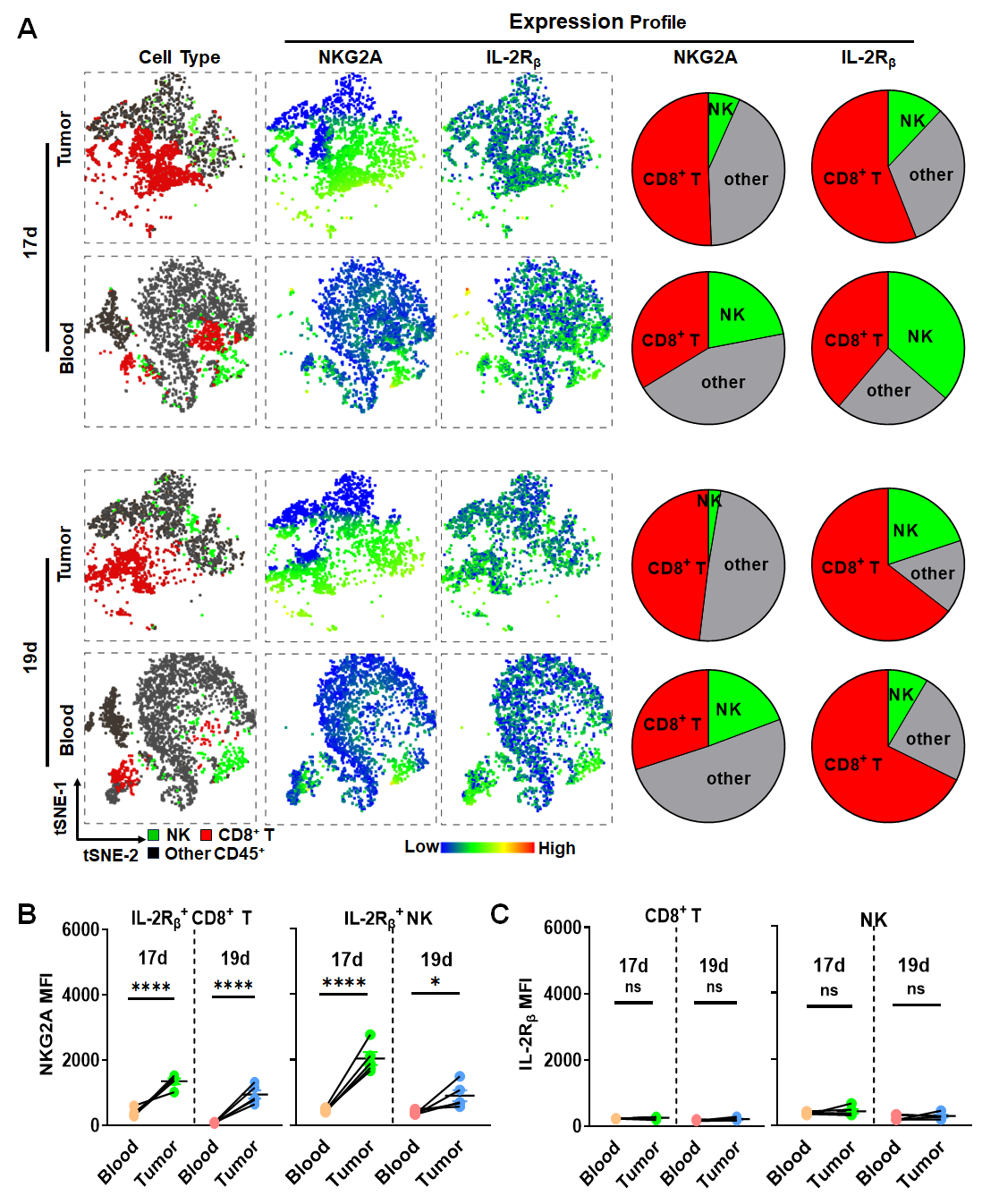
**

**Fig. S3 Expression profiles of NKG2A and IL2Rβ in peripheral and tumor-infiltrated immune cells of mice bearing B16/F1 tumor grafts.** **(A)** tSNE and pie charts indicating NKG2A^+^, and IL-2Rβ^+^ cells in the main CD45^+^ cells from the blood and tumors. **(B)** Expression level of NKG2A in IL-2Rβ^+^ CD8^+^ T and IL-2Rβ^+^ NK cells. **(C)** Expression level of IL-2Rβ in CD8^+^ T and NK cells. Data are represented as mean ± SEM. *p < 0.05, **p < 0.01, ***p < 0.001, ****p < 0.0001, ns: not significant. tSNE dimensionality reduction analysis of CD45^+^ immune subpopulations was performed in mice (N=5) bearing B16/F1 tumor grafts.

**
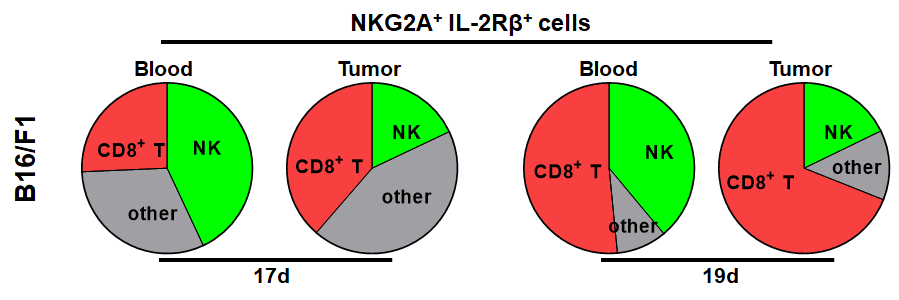
**

**Fig. S4 Pie charts indicating IL-2Rβ^+^NKG2A^+^ cells in the main CD45^+^ cells from the blood and tumor of mice bearing B16/F1 tumor grafts.**

**
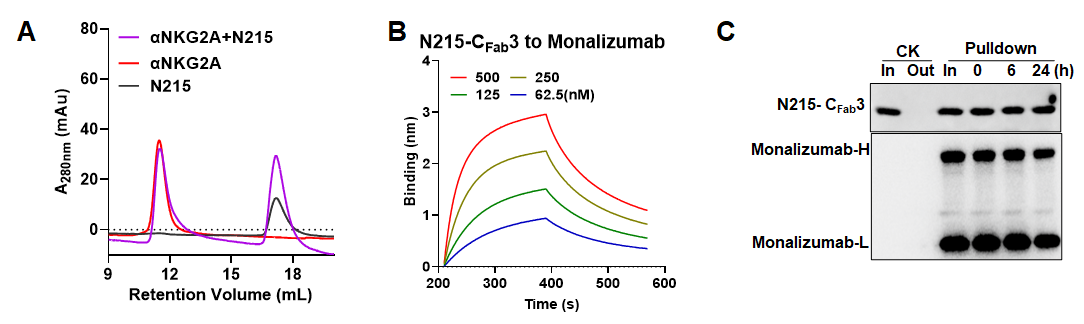
**

**Fig. S5 Coupling of N215 to IgG antibody against human NKG2A. (A)** Binding of N215 to antibody against murine NKG2A (αNKG2A) analyzed by SEC. **(B)** Affinity of N215-C_Fab_3 for antibody against human NKG2A (Monalizumab) measured by biolayer interferometry. **(C)** Pulldown of Monalizumab bound to N215-C_Fab_3 in human serum.


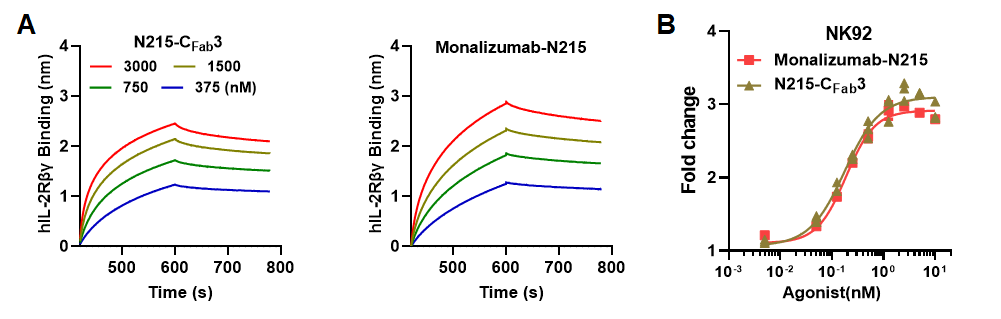


**Fig. S6 Receptor-binding and cell proliferative ability of Monalzumab-N215. (A)** Affinity of N215-C_Fab_3 and Monalizumab-N215 for human IL-2Rβγ measured by biolayer interferometry. **(B)** Proliferation of NK92 stimulated by Monalizumab-N215 and N215-C_Fab_3.


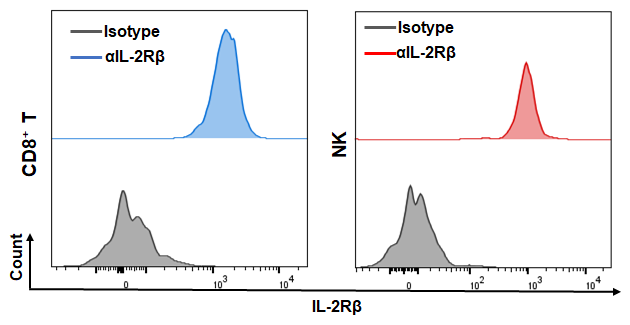


**Fig. S7 Expression of IL-2Rβ in CD8^+^ T and NK cells.**

**
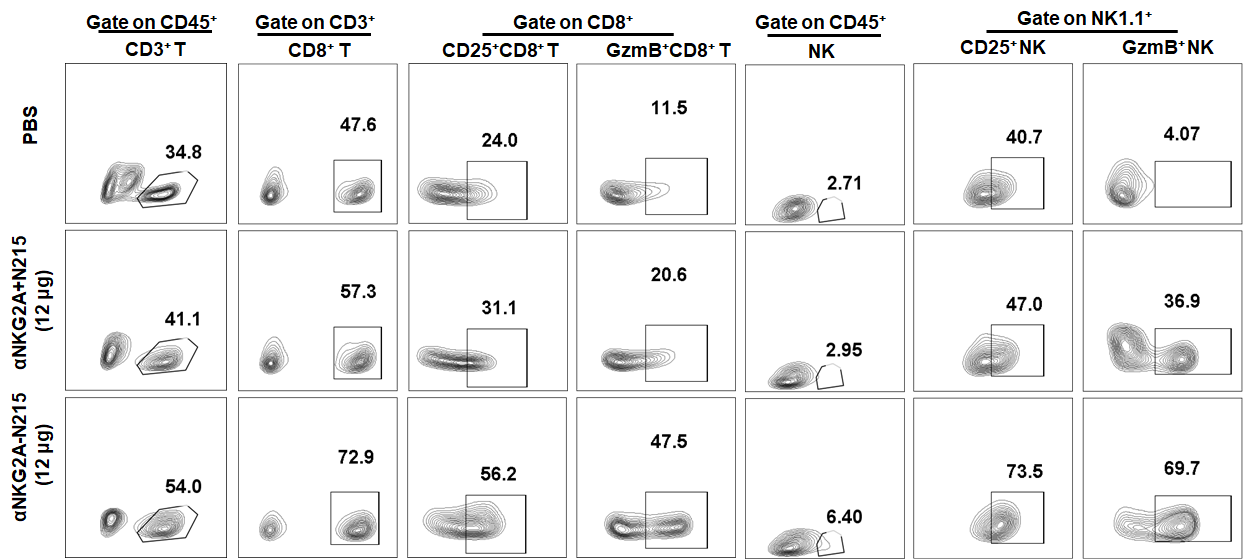
**

**Fig. S8** **Representative flow plots for tumor-infiltrated immune cells in mice bearing MC38 tumor grafts after treatment.**

**
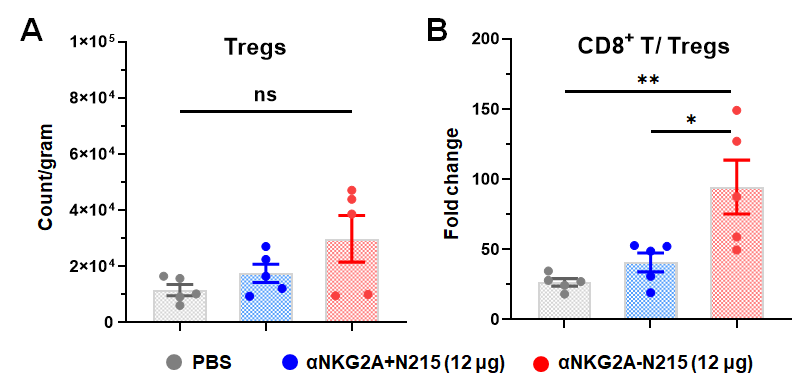
**

**Fig. S9 Tumor-infiltrated Tregs in mice bearing MC38 tumor grafts after treatment. (A)** Counts of tumor-infiltrated Tregs. **(B)** Ratio of CD8^+^ T to Tregs. Data are represented as mean ± SEM. *p < 0.05, **p < 0.01, ns: not significant.

**
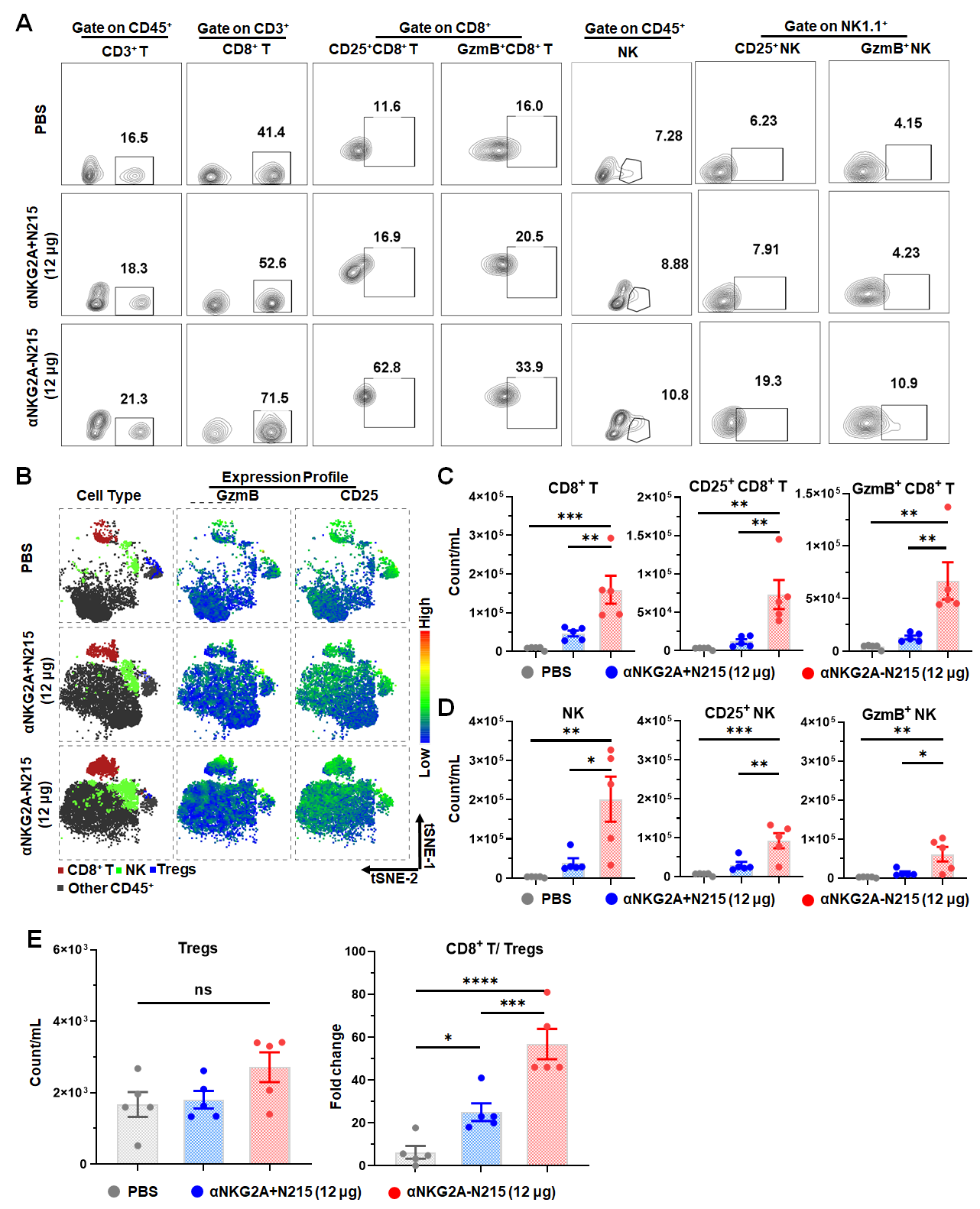
**

**Fig. S10 Peripheral immune cells in mice bearing MC38 tumor grafts after treatment.** **(A)** Representative flow plots. **(B)** tSNE plots indicating GzmB-, CD25-expressing cells. **(C, D, E)** Counts of CD8^+^ T (C), NK (D) cells and Tregs (E). Data are represented as mean ± SEM. *p < 0.05, **p < 0.01, ***p < 0.001, ****p < 0.0001, ns: not significant.

**
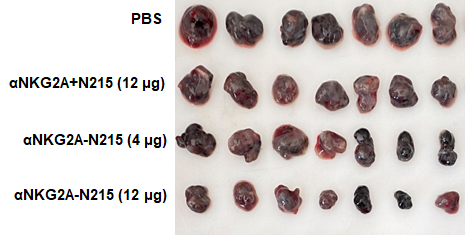
**

**Fig. S11 Photographs of B16/F1 tumors after treatment**.

**
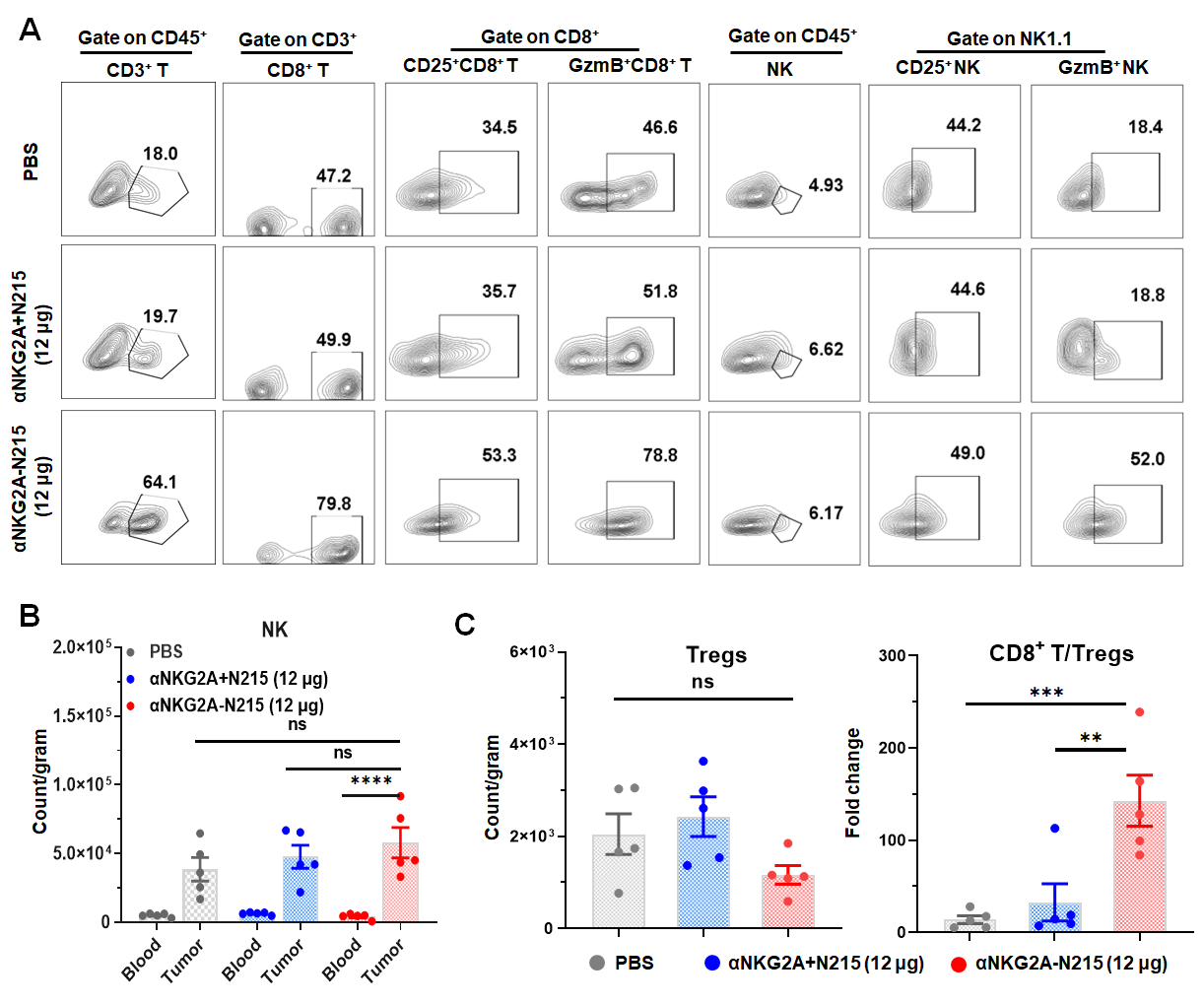
**

**Fig. S12 Tumor-infiltrated immune cells in mice bearing B16/F1 tumor grafts after treatment.** **(A)** Representative flow plots. **(B)** NK cells in the blood and tumors. **(C)** Tumor-infiltrated Tregs and ratio of CD8^+^ T cells to Tregs. Data are represented as mean ± SEM. *p < 0.05, **p < 0.01, ***p < 0.001, ****p < 0.0001, ns: not significant.

**
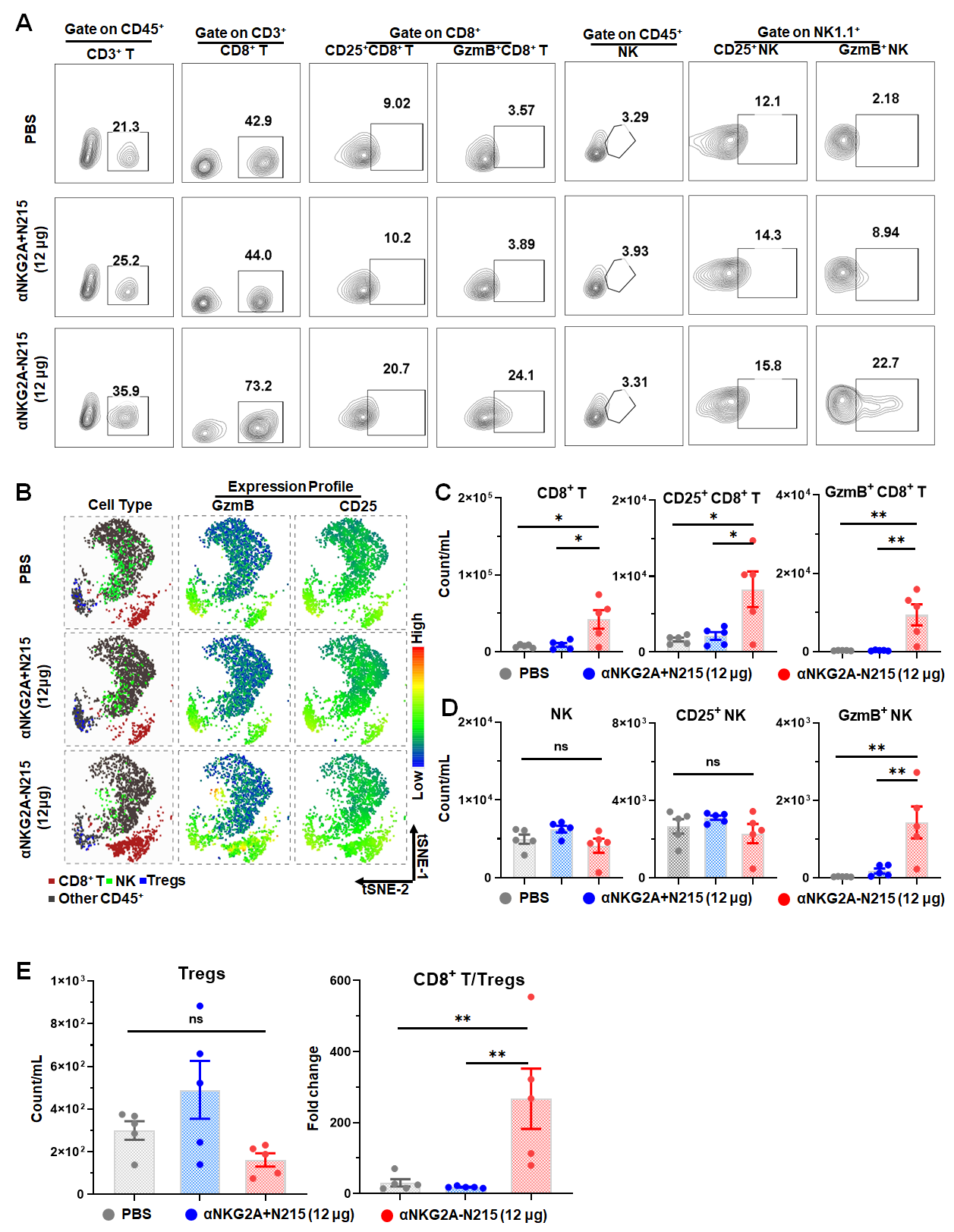
**

**Fig. S13 Peripheral immune cells in mice bearing B16/F1 tumor grafts after treatment. (A)** The representative flow plots. **(B)** tSNE plots indicating GzmB-, and CD25-expressing cells in blood of mice after treatment. **(C, D, E)** CD8^+^ T cells (C), NK cells (D) and Tregs (E) in peripheral blood. Data are represented as mean ± SEM. *p < 0.05, **p < 0.01, ***p < 0.001, ****p < 0.0001, ns: not significant.
